# Intravenous C16 and angiopoietin-1 improve the efficacy of placenta-derived mesenchymal stem cell therapy for EAE

**DOI:** 10.1038/s41598-018-22867-9

**Published:** 2018-03-15

**Authors:** Ke-wei Tian, Yuan-yuan Zhang, Hong Jiang, Shu Han

**Affiliations:** 1 Institute of Anatomy and Cell Biology, Medical College, Zhejiang University, 866 Yuhangtang Road, 310058 Hangzhou, China; 2 Department of Electrophysiology, SirRunRunShaw Hospital, Medical College, Zhejiang University, 310016 Hangzhou, Zhejiang Province China

## Abstract

The placenta has emerged as an attractive source of mesenchymal stem cells (MSCs) because of the absence of ethical issues, non-invasive access, and abundant yield. However, inflammatory cell invasion into grafts negatively impacts the survival and efficacy of transplanted cells. Previous studies have shown that synthetic C16 peptide can competitively block the transmigration of leukocytes into the central nerve system, while angiopoietin-1 (Ang-1) can inhibit inflammation-induced blood vessel leakage and inflammatory cell infiltration in rats with experimental allergic encephalomyelitis (EAE). In this study, we investigated the effects of intravenous administration of C16 and Ang-1 on the efficacy of placenta-derived MSC (PMSC) transplantation in a rat model of EAE. We found that, compared with PMSCs alone, treatment with PMSCs along with intravenously administered C16 and Ang-1 was more effective at ameliorating demyelination/neuronal loss and neurological dysfunction, reducing inflammatory cell infiltration, perivascular edema, and reactive astrogliosis (p < 0.05). Mechanistic studies revealed that intravenous C16 and Ang-1 increased PMSC engraftment in the central nervous system and promoted expression of the neurotropic proteins brain-derived neurotrophic factor, growth-associated protein 43, and p75 neurotrophin receptor as well as the neuronal-glial lineage markers neurofilament protein 200 and myelin basic protein in the engrafted PMSCs.

## Introduction

The autoimmune disease multiple sclerosis (MS) affects the central nervous system (CNS) and has great socio-economic impact in developed countries^1^. In MS, the immune system attacks the protective sheath (myelin) of nerve fibers, eventually leading to permanent nerve damage and neurological disability^2^. Mesenchymal stem cells (MSCs) have demonstrated immunoregulatory and neuroprotective functions in animal models of MS, and thus, are considered a new potential therapeutic modality for this disease^3–5^. Apart from their high proliferation and differentiation potential, embryonic MSCs (EMSCs) have been shown to exhibit superior immunoregulatory properties, and therefore, outperform bone marrow MSCs in the treatment of experimental allergic encephalomyelitis (EAE), a common model of MS^6–8^. However, the application of EMSCs is limited by ethical concerns. In recent years, placenta-derived MSCs (PMSCs) have emerged as an attractive alternate source of MSCs for their lack of ethical issues, non-invasive access, and abundant yield^9^. In a recent study, transplanted PMSCs were shown to reduce disease severity and improve survival in a mouse EAE model, presumably through the release of the anti-inflammatory protein tumor necrosis factor alpha (TNF-α)-stimulated gene/protein 6 (TSG-6)^10^.

Increased blood–brain barrier (BBB) permeability and infiltration of inflammatory cells into the CNS lead to demyelination and neuronal dysfunction in EAE^11^. In MSC treatment of EAE, inflammatory factors such as nuclear factor kappa-light chain-enhancer of activated B cells (NF-κB), tumor necrosis factor alpha (TNF-α), and cyclooxygenase 2 (COX-2) in the inflamed CNS microenvironment can negatively impact the survival of grafted cells^12^. Thus, blocking inflammatory cell infiltration should protect not only the neurons within the CNS of transplant recipients but also the transplanted MSCs themselves. Angiopoietin-1 (Ang-1), an endothelial growth factor, is well documented to promote and maintain vascular maturation, homeostasis, and integrity^13^. It has been shown to inhibit inflammation-induced blood vessel leakage and inflammatory cell infiltration in a rat model of EAE^14^. C16 is a synthetic peptide that selectively binds the αvβ3 and αvβ1 integrins expressed on endothelial cells, and this binding has been shown to inhibit inflammatory cell transmigration by blocking leukocyte–endothelial interaction^15^. Furthermore, C16 and Ang-1 have been reported to work synergistically to mitigate vascular leakage and inflammation and protect against demyelination and axonal loss in rats with EAE^14^.

In the present study, we examined the effects of intravenous C16 and Ang-1 on the efficacy of PMSC transplantation for treating EAE in a rat model. The neurological functions, CNS infiltration of inflammatory cells, perivascular edema, white matter demyelination, axonal loss, neuronal apoptosis, and reactive astrogliosis were evaluated. The homing of transplanted PMSCs to the CNS as well as the expression of the neurotrophic proteins brain-derived neurotrophic factor (BDNF), growth-associated protein 43 (GAP-43), p75 neurotrophin receptor (p75NTR) and the neuronal-glial lineage markers neurofilament protein 200 (NF-200) and myelin basic protein (MBP) in the engrafted PMSCs were examined.

## Results

### Intravenous C16 and Ang-1 enhanced the efficacy of PMSC therapy for preventing neurological dysfunctions in rats with EAE

**Figure 1:**
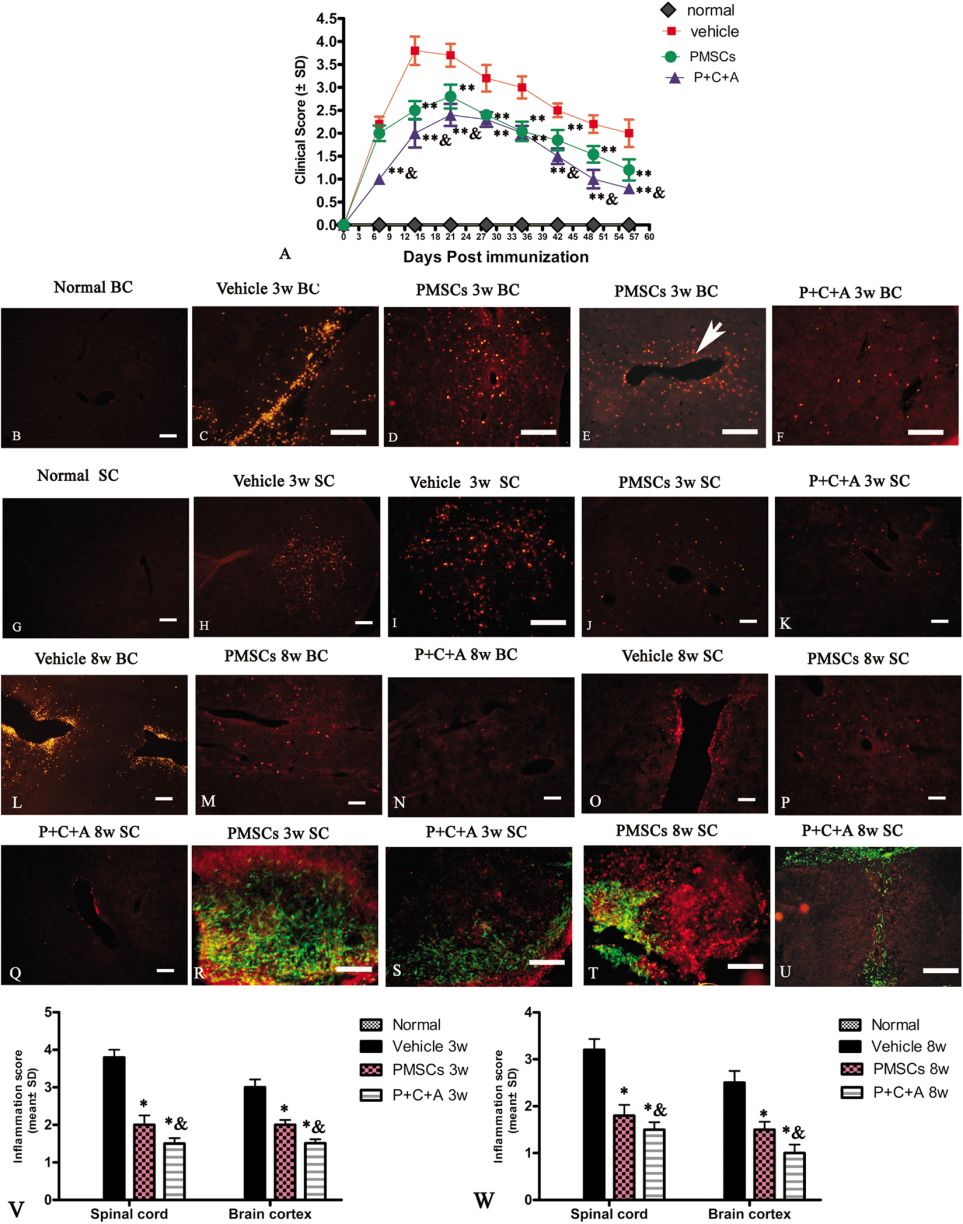
Intravenous C16 and Ang-1 enhanced the efficacy of PMSC therapy for reducing inflammatory cell infiltration and disease progression in the rat EAE model. (**A**) Clinical scoring of the severity of EAE symptoms post-injection (pi). n = 10, **P < 0.01 vs. vehicle (PMSCs vs. vehicle: 1 week pi, ES = 1.18, *P* = 0.28; 2 weeks pi, ES = 8.98, *P* < 0.0001; 3 weeks pi, ES = 6.72, *P* = 0.0003; 4 weeks pi, ES = 8.03, *P* = 0.00023; 5 weeks pi, ES = 9.27, *P* < 0.0001; 6 weeks pi, ES = 8.87, *P* = 0.000332; 7 weeks pi, ES = 9.9, *P* = 0.0002; 8 weeks pi, ES = 5.12, *P* = 0.00097. P + C + A vs. vehicle: 1 week pi, ES = 24.75, *P* < 0.0001; 2 weeks pi, ES = 9.067, *P* < 0.0001; 3 weeks pi, ES = 10.84, *P* < 0.0001; 4 weeks pi, ES = 18.11, *P* = 0.00015; 5 weeks pi, ES = 11.93, *P* < 0.0001; 6 weeks pi, ES = 18.7, *P* < 0.0001; 7 weeks pi, ES = 15.61, *P* < 0.0001; 8 weeks pi, ES = 10.66, *P* = 0.000262), ^&^*P* < 0.05 vs. PMSCs (1 week pi, ES = 24.75, *P* < 0.0001; 2 weeks pi, ES = 3.2, *P* = 0.013; 3 weeks pi, ES = 3.36, *P* = 0.014; 4 weeks pi, ES = 2.45, *P* = 0.15; 5 weeks pi, ES = 0.39, *P* = 0.42; 6 weeks pi, ES = 4.65, *P* = 0.0099; 7 weeks pi, ES = 7.39, *P* = 0.0001; 8 weeks pi, ES = 5.61, *P* = 0.0061). (**B**–**Q**) Representative immunofluorescence staining images showing infiltration of macrophages (CD68^+^) into the brain cortex and spinal cord of rats at 3 and 8 weeks pi. SC denotes transverse sections through the anterior horn of the lumbar spine, and BC denotes coronal sections of the motor cortex. Scale bar = 100 μm. (**V**,**W**) Scoring of the severity of inflammatory cell infiltration at 3 (**V**) and 8 weeks (**W**) pi. n = 5, *P < 0.05 vs. vehicle (PMSCs vs. vehicle at 3 weeks pi: ES = 17.87, *P* < 0.0001 in SC; ES = 17.15, *P* < 0.0001 in BC. P + C + A vs. vehicle at 3 weeks pi: E = 36.46, *P* < 0.0001 in SC; ES = 28.15, *P* < 0.0001 in BC. PMSCs vs. vehicle at 8 weeks pi: ES = 14.31, *P* < 0.0001 in SC; ES = 10.72, *P* < 0.0001 in BC. P + C + A vs. vehicle at 8 weeks pi: ES = 21.84, *P* < 0.0001 in SC; ES = 15.88, *P* < 0.0001 in BC), ^&^*P* < 0.05 vs. PMSCs (3 weeks pi in SC, ES = 5.82, *P* = 0.0028; 3 weeks pi in BC, ES = 17.98, *P* < 0.0001; 8 weeks pi in SC, ES = 4.34, *P* = 0.017; 8 weeks pi in BC, ES = 8.87, *P* = 0.00058).

Neurological dysfunctions in rats with EAE started 1 week post immunization (pi) (clinical scores > 2) and quickly progressed to the peak level (clinical scores ~3.7) by 2 weeks pi (Fig. 1A). After that, the rats underwent spontaneous recovery and the clinical scores returned to 2 by 8 weeks pi (Fig. 1A). Rats treated with PMSCs only also exhibited symptoms as early as 1 week pi, whereas those treated with PMSCs plus intravenous C16 and Ang-1 did not show neurological dysfunction until 2 weeks pi (Fig. 1A). The symptoms peaked at 3 weeks pi in both the PMSCs and P + C + A groups, which was 1 week later than in the vehicle-treated group (Fig. 1A). The clinical scores of rats in the PMSCs group were significantly lower than those in the vehicle-treated group from 2 to 8 weeks pi, and the scores were lower still in the P + C + A group from 1 to 3 weeks pi (the onset stage) and from 6 to 8 weeks pi (the recovery stage; Fig. 1A, *P* < 0.05).

Changes in cortical somatosensory evoked potential (CSEP) and cortical motor evoked potential (CMEP) have been commonly used to assess the level of neural damage in MS patients^16–20^. Compared with normal rats, rats in which EAE was induced displayed prolonged latency to waveform initiation and lower peak amplitudes for both CSEP and CMEP at 3 and 8 weeks pi (Table 1, Fig. S1), indicating a slower speed of conduction and loss of functioning nerve fibers, respectively, in these rats. These EAE-associated electrophysiological disturbances were significantly attenuated by treatment with PMSCs and PMSCs plus intravenous C16 and Ang-1 (Table 1, Fig. S1; *P* < 0.05).

**Table 1:** Intravenous C16 and Ang-1 enhanced the efficacy of PMSC therapy for restoring CSEP and CMEP latency and amplitude in rats with EAE.

3 weeks pi	CSEP Latency (ms)		
Group	N	P	Wave amplitude (V_mean_ ± SD)
Normal	11.6 ± 0.20** (ES = 8.14, *P* = 0.003)	16.22 ± 0.24** (ES = 6.59, *P* = 0.0027)	2.15 ± 0.36* (ES = 10.29, *P* = 0.011)
Vehicle	18.38 ± 0.89	25.32 ± 1.15	0.78 ± 0.06
PMSCs	13.44 ± 1.54** (ES = 0.76, *P* = 0.004)	19.45 ± 0.85** (ES = 2.87, *P* = 0.001)	2.34 ± 0.68* (ES = 3.35, *P* = 0.03)
P + C + A	12.08 ± 0.99** (ES = 3.55, *P* = 0.0006; vs. PMSCs: ES = 0.4, *P* = 0.13)	17.31 ± 0.42**^&^ ES = 5.34, *P* = 0.0001; vs. PMSCs: ES = 2.38, *P* = 0.009)	2.07 ± 0.33* (ES = 11.47, *P* = 0.0109; vs. PMSCs: ES = 0.47, *P* = 0.28)
**3 weeks pi**	**CMEP Latency (ms)**		
**Group**		**Wave amplitude (V**_**mean**_ ** ± SD)**	
Normal	5.2 ± 0.14** (ES = 9.09, *P* = 0.002)	4.56 ± 0.23** (ES = 71.15, *P* < 0.0001)	
Vehicle	13.24 ± 0.93	0.54 ± 0.06	
PMSCs	5.81 ± 0.22** (ES = 8.13, *P* < 0.0001)	4.45 ± 1.11* (ES = 3.16, *P* = 0.012)	
P + C + A	5.83 ± 0.34** (ES = 7.55, *P* = 0.0001; vs. PMSCs: ES = 0.12, *P* = 0.46)	5.69 ± 0.76** (ES = 8.86, *P* = 0.004; vs. PMSCs: ES = 0.69, *P* = 0.09)	
**8 weeks pi**	**CSEP Latency (ms)**		
**Groups**	**N**	**P**	**Wave amplitude (V**_**mean**_ ** ± SD)**
Normal	11.22 ± 0.94** (ES = 2.53, *P* = 0.0002)	16.76 ± 1.08** (ES = 3.34, *P* = 0.0001)	2.86 ± 0.33** (ES = 16.26, *P* = 0.005)
Vehicle	27.22 ± 2.33	32.98 ± 1.92	1.01 ± 0.07
PMSCs	16.85 ± 1.68** (ES = 2.16, *P* = 0.0017)	22.41 ± 1.48** (ES = 1.8, *P* = 0.0008)	2.69 ± 0.36** (ES = 12.49, *P* = 0.0078)
P + C + A	14.32 ± 1.35** (ES = 1.77, *P* = 0.0006; vs. PMSCs: ES = 0.54, *P* = 0.06)	19.01 ± 1.78**^&^ (ES = 2.04, *P* = 0.0004; vs. PMSCs: ES = 0.63, *P* = 0.03)	2.50 ± 0.14** (ES = 60.82, *P* < 0.0001; vs. PMSCs: ES = 1.27, *P* = 0.22)
**8 weeks pi**	**CMEP Latency (ms)**		
**Groups**		**Wave amplitude (V**_**mean**_ ** ± SD)**	
Normal	5.32 ± 0.43** (ES = 52.22, *P* < 0.0001)	7.39 ± 1.31** (ES = 4.19, *P* = 0.005)	
Vehicle	17.28 ± 0.21	0.17 ± 0.09	
PMSCs	6.34 ± 0.88** (ES = 13.37, *P* < 0.0001)	4.36 ± 1.16* (ES = 3.1, *P* = 0.012)	
P + C + A	5.52 ± 1.45** (ES = 5.48, *P* = 0.003; vs. PMSCs: ES = 0.29, *P* = 0.22)	4.73 ± 0.55** (ES = 14.68, *P* = 0.0002; vs. PMSCs: ES = 0.22, *P* = 0.21)	

*P < 0.05, **P < 0.01 vs. vehicle; ^&^*P* < 0.05 vs. PMSCs. N, negative deflection; P, positive deflection; pi, post-immunization.

### Intravenous C16 and Ang-1 enhanced the efficacy of PMSC therapy for inhibiting inflammatory cell infiltration in rats with EAE

Immunostaining of tissues collected 3 weeks pi showed diffuse CNS infiltration of CD68^+^ cells around blood vessels, throughout brain tissue and spinal cord parenchyma, and below the meninges (Fig. 1C,H–I,L,O). The perivascular and parenchymal infiltrates at 3 and 8 weeks pi were significantly reduced by PMSC transplantation (Fig. 1D,E,J,M,P) and further decreased by treatment with PMSCs plus intravenous C16 and Ang-1 (Fig. 1F,K,N,Q). These results were confirmed by inflammatory scores at 3 and 8 weeks pi (Fig. 1V,W; *P* < 0.05). CD68 was not detected in the engrafted PMSCs; however, significant CD68-positive cell infiltrates were detected in the areas surrounding the grafts and in the central region of the graft cell mass. Cell invasion into the grafts was less severe in the P + C + A group than in the PMSCs group (Fig. 1R–T).

### Intravenous C16 and Ang-1 enhanced the anti-inflammatory and anti-astrogliosis effects of PMSCs in rats with EAE

**Figure 2:**
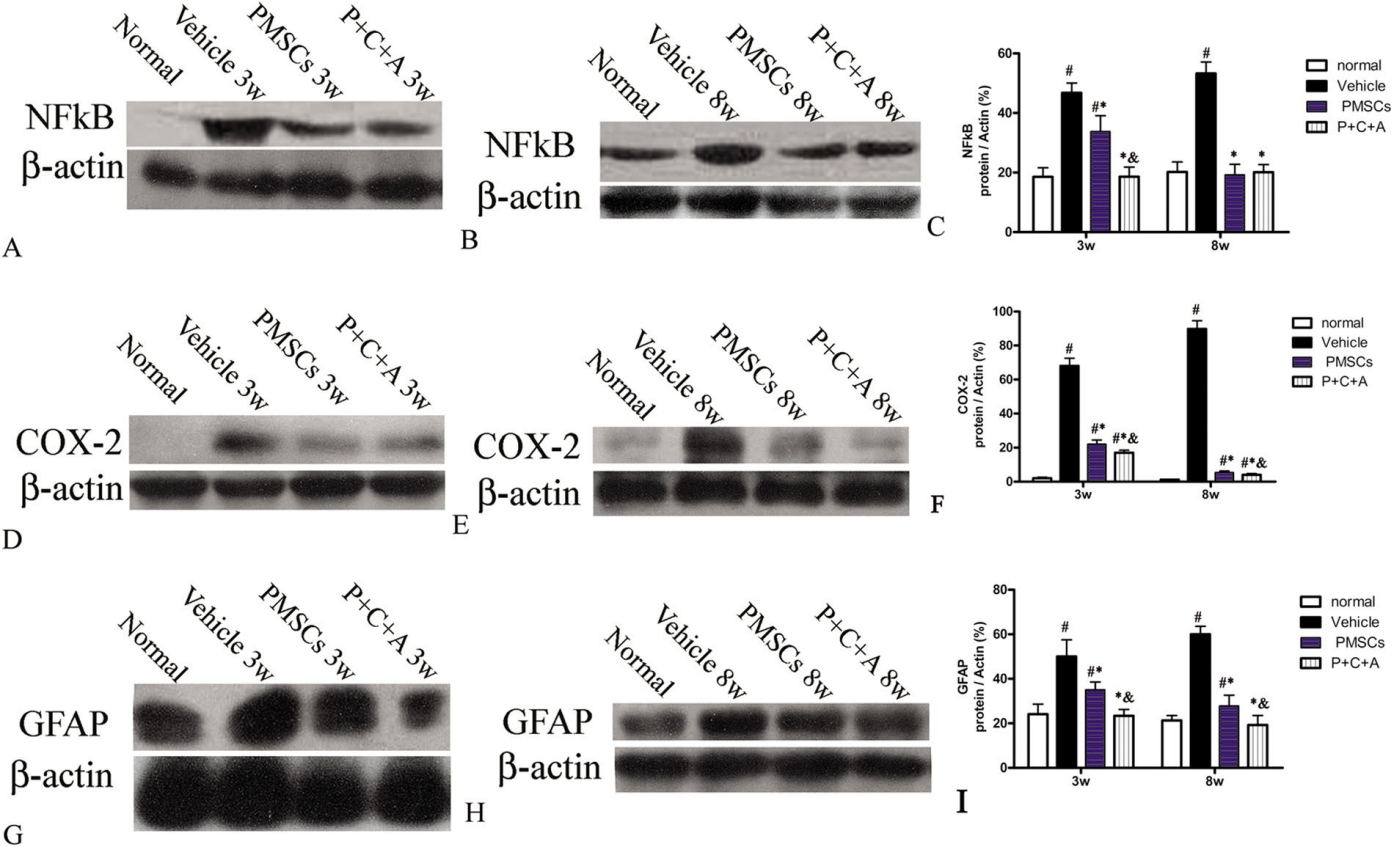
Intravenous C16 and Ang-1 enhanced the anti-inflammatory and anti-astrogliosis effects of PMSCs in the brain cortex of rats with EAE. Levels of NF-κB (**A**–**C**), COX-2 (**D**–**F**) and GFAP (**G**–**I**) in the brain cortex at 3 and 8 weeks pi were determined by western blotting. n = 5, ^#^*P* < 0.05 vs. normal, ^*^P < 0.05 vs. vehicle, ^&^*P* < 0.05 vs. PMSCs. (**C**) At 3 weeks pi, PMSCs vs. vehicle: ES = 0.33, *P* = 0.0008; P + C + A vs. vehicle: ES = 1.34, *P* = 0.0003; P + C + A vs. PMSCs: ES = 0.4, *P* = 0.0003. At 8 weeks pi, PMSCs vs. vehicle: ES = 1.24, *P* < 0.0001; P + C + A vs. vehicle: ES = 1.53, *P* < 0.0001; P + C + A vs. PMSCs: ES = 0.05, *P* = 0.33. (**F**) At 3 weeks pi, PMSCs vs. vehicle: ES = 1.7, *P* < 0.0001; P + C + A vs. vehicle: ES = 2.22, *P* < 0.0001; P + C + A vs. PMSCs: ES = 0.56, *P* = 0.003. At 8 weeks pi, PMSCs vs. vehicle: ES = 3.53, *P* < 0.0001; P + C + A vs. vehicle: ES = 3.61, *P* < 0.0001; P + C + A vs. PMSCs: ES = 1.04, *P* = 0.02. (**I**) At 3 weeks pi, PMSCs vs. vehicle: ES = 0.22, *P* = 0.002; P + C + A vs. vehicle: ES = 0.41, *P* < 0.0001; P + C + A vs. PMSCs: ES = 0.55, *P* = 0.0002. At 8 weeks pi, PMSCs vs. vehicle: ES = 0.9, *P* < 0.0001; P + C + A vs. vehicle: ES = 1.31, *P* < 0.0001; P + C + A vs. PMSCs: ES = 0.21, *P* = 0.009.

**Figure 3:**
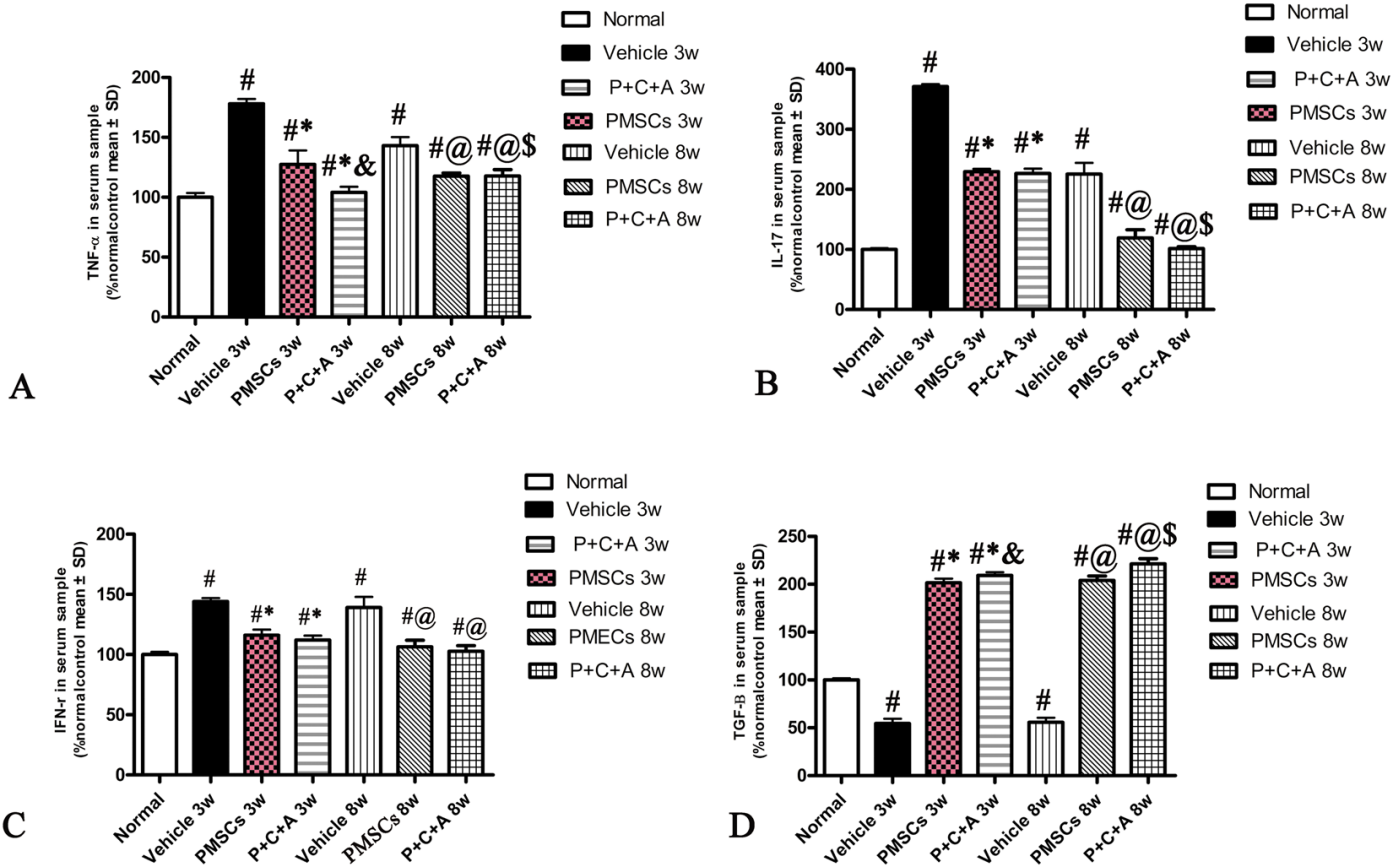
Intravenous C16 and Ang-1 enhanced the anti-inflammatory effects of PMSCs in rats with EAE as indicated in serum levels of TNF-α (**A**), IL-17 (**B**), IFN-γ (**C**) and TGF-β (**D**) at 3 and 8 weeks pi by ELISA. n = 5, ^#^*P* < 0.05 vs. normal, ^*^P < 0.05 vs. vehicle at 3 weeks pi, ^**&**^*P* < 0.05 vs. PMSCs at 3 weeks pi, ^*@*^*P* < 0.05 vs. vehicle at 8 weeks pi, ^$^*P* < 0.05 vs. PMSCs at 8 weeks pi. (**A**) At 3 weeks pi, PMSCs vs. vehicle: ES = 0.33, *P* = 0.0001; P + C + A vs. vehicle: ES = 1.98, *P* < 0.0001; P + C + A vs. PMSCs: ES = 0.15, *P* = 0.004. AT 8 weeks pi, PMSCs vs. vehicle: ES = 0.45, *P* = 0.0003; P + C + A vs. vehicle: ES = 0.45, *P* < 0.0001; P + C + A vs. PMSCs: ES = 0.28, *P* = 0.003. (**B**) At 3 weeks pi, PMSCs vs. vehicle: ES = 3.895, *P* < 0.0001; P + C + A vs. vehicle: ES = 1.86, *P* < 0.0001; P + C + A vs. PMSCs: ES = 0.04, *p* = 0.22. AT 8 weeks pi, PMSCs vs. vehicle: ES = 0.2, *P* < 0.0001; P + C + A vs. vehicle: ES = 0.34, *P* < 0.0001; P + C + A vs. PMSCs: ES = 0.09, *P* = 0.03. (**C**) At 3 weeks pi, PMSCs vs. vehicle: ES = 0.9, *P* < 0.0001; P + C + A vs. vehicle: ES = 1.51, *P* < 0.0001); P + C + A vs. PMSCs: ES = 0.1, *P* = 0.09; At 8 weeks pi, PMSCs vs. vehicle: ES = 0.3, *P* = 0.028; P + C + A vs. vehicle: ES = 0.36, *P* < 0.0001; P + C + A vs. PMSCs: ES = 0.07, *P* = 0.15. (**D**) At 3 weeks pi, PMSCs vs. vehicle: ES = 3.22, *P* < 0.0001; P + C + A vs. vehicle: ES = 4, *P* < 0.0001; P + C + A vs. PMSCs: ES = 0.24, *P* = 0.008. At 8 weeks pi, PMSCs vs. vehicle: ES = 3.47, *P* < 0.0001; P + C + A vs. vehicle: ES = 3.31, *P* < 0.0001; P + C + A vs. PMSCs: ES = 0.36, *P* = 0.0003.

At 3 and 8 weeks pi, expression of the pro-inflammatory factors NF-κB and COX-2 in the brain cortex and spinal cords was analyzed by RT-PCR (Fig. SP2), western blotting (Fig. 2A–F) and immunostaining (Figs S4, S5). In addition, serum TNF-α, IL-17, IFN-γ, and TGF-β levels were measured by ELISA (Fig. 3A–D). The levels of NF-κB, COX-2, TNF-α, IL-17, and IFN-γ were significantly higher in rats with EAE than in normal rats (Figs 2, 3, S2–5). The increases in these pro-inflammatory factors in rats with EAE were significantly reduced by PMSC therapy (*P* < 0.05) and further decreased by treatment with PMSCs plus intravenous C16 and Ang-1 (*P* < 0.05) expect for IFN-γ (Figs 2, 3, S2–5). In contrast, the serum TGF-β level was slightly lower in rats with EAE than in normal rats at 3 and 8 weeks pi (Fig. 3D, P < 0.05). PMSC transplantation restored the TGF-β levels in rats with EAE, and treatment with PMSCs plus intravenous C16 and Ang-1 further increased the TGF-β levels (Fig. 3D, *P* < 0.05).

**Figure 4:**
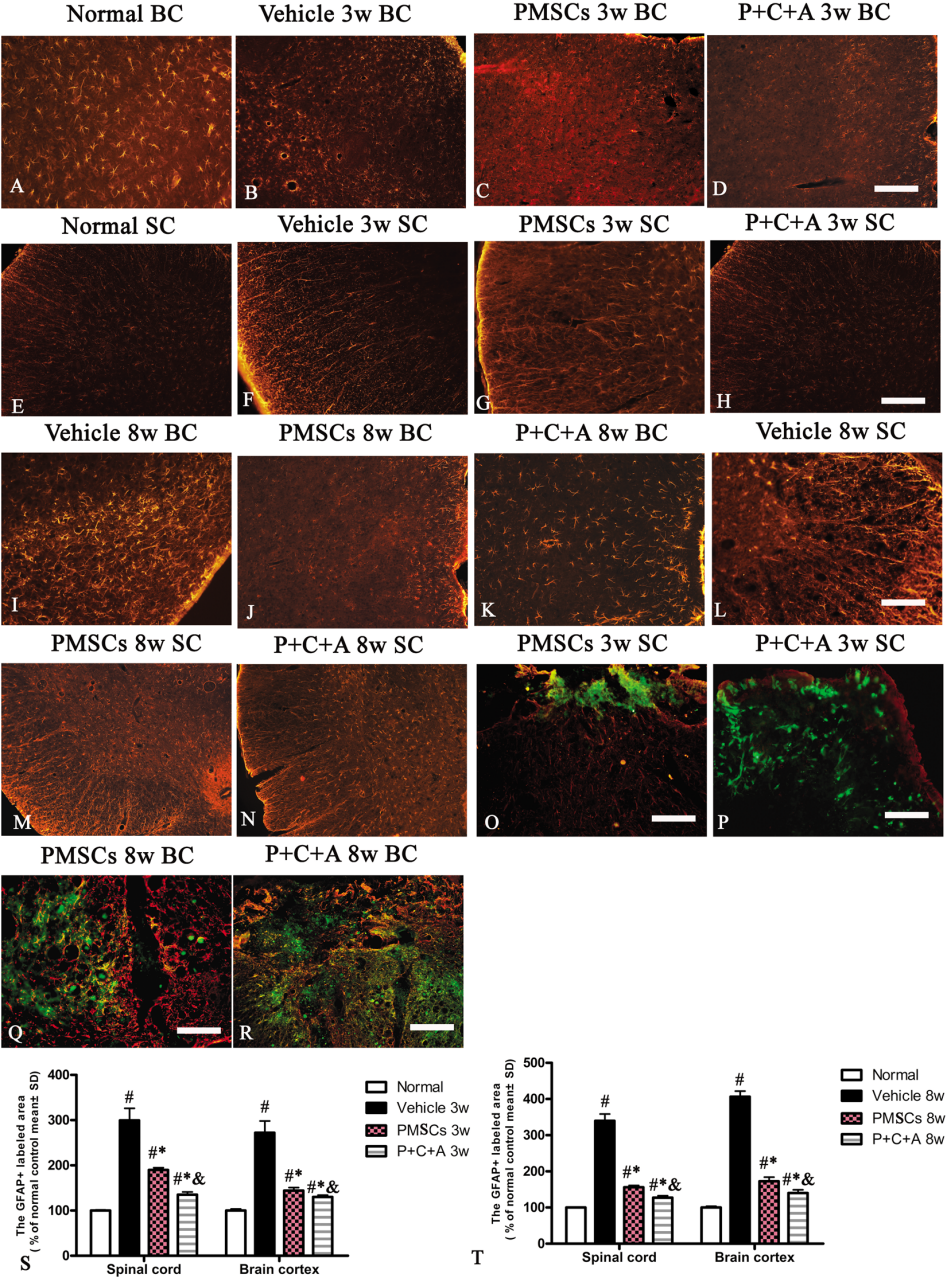
Intravenous C16 and Ang-1 enhanced the efficacy of PMSC therapy for inhibiting reactive astrogliosis in the EAE rat model. (**A**–**N**) Immunofluorescence staining of brain cortex and spinal cord specimens for the astrocyte marker GFAP (red) at 3 and 8 weeks pi. SC denotes transverse sections through the anterior horn of the lumbar spine, and BC denotes coronal sections of the motor cortex. (**O**–**R**) Immunofluorescence staining of the PMSC grafts (green) for GFAP (red). Scale bar = 100 μm. (**S**,**T**) Relative areas of GFAP staining at 3 (**S**) and 8 (**T**) weeks pi. n = 5, ^#^*P* < 0.05 vs. normal, ^*^P < 0.05 vs. vehicle, ^&^*P* < 0.05 vs. PMSCs. (**S**) In SC, PMSCs vs. vehicle: ES = 0.15, *P* = 0.0003; P + C + A vs. vehicle: ES = 0.22, *P* < 0.0001; P + C + A vs. PMSCs: ES = 0.9, *P* < 0.0001. In BC, PMSCs vs. vehicle: ES = 0.18, *P* = 0.0002; P + C + A vs. vehicle: ES = 0.21, *P* = 0.0001; P + C + A vs. PMSCs: ES = 0.25, *P* = 0.0016. (**T**) In SC, PMSCs vs. vehicle: ES = 0.49, *P* < 0.0001; P + C + A vs. vehicle: ES = 0.56, *P* < 0.0001; P + C + A vs. PMSCs: ES = 0.52, *P* < 0.0001. In BC, PMSCs vs. vehicle: ES = 0.64, *P* < 0.0001; P + C + A vs. vehicle: ES = 0.85, *P* < 0.0001; P + C + A vs. PMSCs: ES = 0.16, *P* = 0.0005.

Astrocyte proliferation in response to neuronal injury in EAE (reactive astrogliosis) drives CNS inflammation and can lead to the formation of glial scars at the lesion site, which in turn inhibits axonal regeneration^21^. In this study, we assessed the expression and distribution of the specific astrocyte marker GFAP in the brain cortex and spinal cord by immunofluorescence staining. The results revealed astrocyte proliferation from 3 weeks pi (Fig. 4B,F), with noticeable glial scar formation at 8 weeks pi in rats with EAE (Fig. 4I,L) but not in normal rats (Fig. 4A,E). Astrocyte proliferation was significantly inhibited by treatment with PMSCs alone or in combination with intravenous C16 and Ang-1 at both 3 (Fig. 4C,D,G,H) and 8 weeks (Fig. 4J,K,M,N) PI. Moreover, P + C + A rats exhibited significantly reduced astrocyte proliferation compared with PMSCs rats at 8 weeks pi (Fig. 4S,T; *P* < 0.05). These results were confirmed by western blot (Fig. 4G–I) and RT-PCR (Fig. SP2) analysis of GFAP expression in the brain cortex.

GFAP was not detected in the engrafted PMSCs; however, astrocyte proliferation was observed in areas circumjacent to the grafts, which, in turn, obstructed the transmigration of PMSCs (Fig. 4O–R). Consequently, the P + C + A group exhibited more efficient PMSC homing to the CNS compared with the PMSCs group (Fig. 4O–R).

### Intravenous C16 and Ang-1 enhanced the efficacy of PMSC therapy for preventing demyelination, vascular leakage, and neuronal loss in rats with EAE

**Figure 5:**
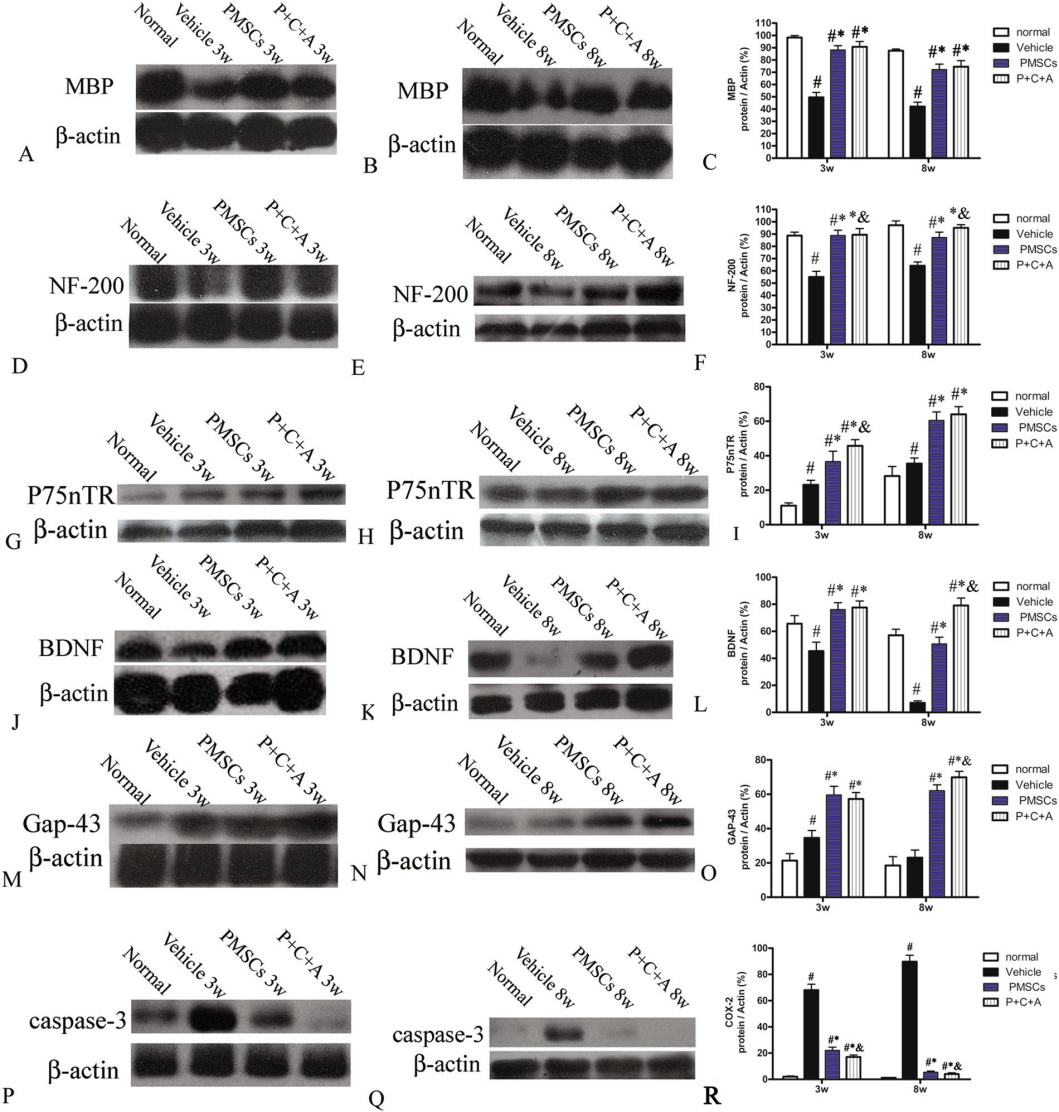
Protein expression of MBP (**A**–**C**), NF-200 (**D**–**F**), p75NTR (**G**–**I**), BDNF (**J**–**L**), GAP-43 (**M**–**O**) and caspase-3 (**P**–**R**) in the brain cortex at 3 and 8 weeks pi by western blotting. n = 5. ^#^*P* < 0.05 vs. normal, ^*^P < 0.05 vs. vehicle, ^&^*P* < 0.05 vs. PMSCs. (**C**) At 3 weeks pi, PMSCs vs. vehicle: ES = 1.34, *P* < 0.0001; P + C + A vs. vehicle: ES = 1.17, *P* < 0.0001; P + C + A vs. PMSCs ES = 0.08, *P* = 0.17. At 8 weeks pi, PMSCs vs. vehicle: ES = 0.9, *P* < 0.0001; P + C + A vs. vehicle: ES = 0.85, *P* < 0.0001; P + C + A vs. PMSCs: ES = 0.05, *P* = 0.22. (**F**) At 3 weeks pi, PMSCs vs. vehicle: ES = 0.72, *P* < 0.0001; P + C + A vs. vehicle: ES = 0.75, *P* < 0.0001; P + C + A vs. PMSCs: ES = 0.13, *P* = 0.047. At 8 weeks pi, PMSCs vs. vehicle: ES = 0.75, *P* < 0.0001; P + C + A vs. vehicle: ES = 1.87, *P* < 0.0001; P + C + A vs. PMSCs: ES = 0.3, *P* = 0.004. (**I**) At 3 weeks pi, PMSCs vs. vehicle: ES = 0.31, *P* = 0.0009; P + C + A vs. vehicle: ES = 1.54, *P* < 0.0001; P + C + A vs. PMSCs: ES = 0.19, *P* = 0.009. At 8 weeks pi, PMSCs vs. vehicle: ES = 0.69, *P* < 0.0001; P + C + A vs. vehicle: ES = 0.85, *P* < 0.0001; P + C + A vs. PMSCs: ES = 0.03, *P* = 0.3. (**L**) At 3 weeks pi, PMSCs vs. vehicle: ES = 0.42, *P* < 0.0001; P + C + A vs. vehicle: ES = 0.49, *P* < 0.0001; P + C + A vs. PMSCs: ES = 0.03, *P* = 0.31. At 8 weeks pi, PMSCs vs. vehicle: ES = 1.57, *P* < 0.0001; P + C + A vs. vehicle: ES = 2.18, *P* < 0.0001; P + C + A vs. PMSCs: ES = 0.51, *P* < 0.0001. (**O**) At 3 weeks pi, PMSCs vs. vehicle: ES = 0.57, *P* < 0.0001; P + C + A vs. vehicle: ES = 0.68, *P* < 0.0001; P + C + A vs. PMSCs: ES = 0.06, *P* = 0.21. At 8 weeks pi, PMSCs vs. vehicle: ES = 1.18, *P* < 0.0001; P + C + A vs. vehicle: ES = 1.46, *P* < 0.0001; P + C + A vs. PMSCs: ES = 0.32, *P* = 0.004. (**R**) At 3 weeks pi, PMSCs vs. vehicle: ES = 1.34, *P* < 0.0001; P + C + A vs. vehicle: ES = 2.42, *P* < 0.0001; P + C + A vs. PMSCs: ES = 1.73, *P* < 0.0001. At 8 weeks pi, PMSCs vs. vehicle: ES = 3.56, *P* < 0.0001; P + C + A vs. vehicle: ES = 5.19, *P* < 0.0001; P + C + A vs. PMSCs: ES = 0.37, *P* = 0.04.

**Figure 6:**
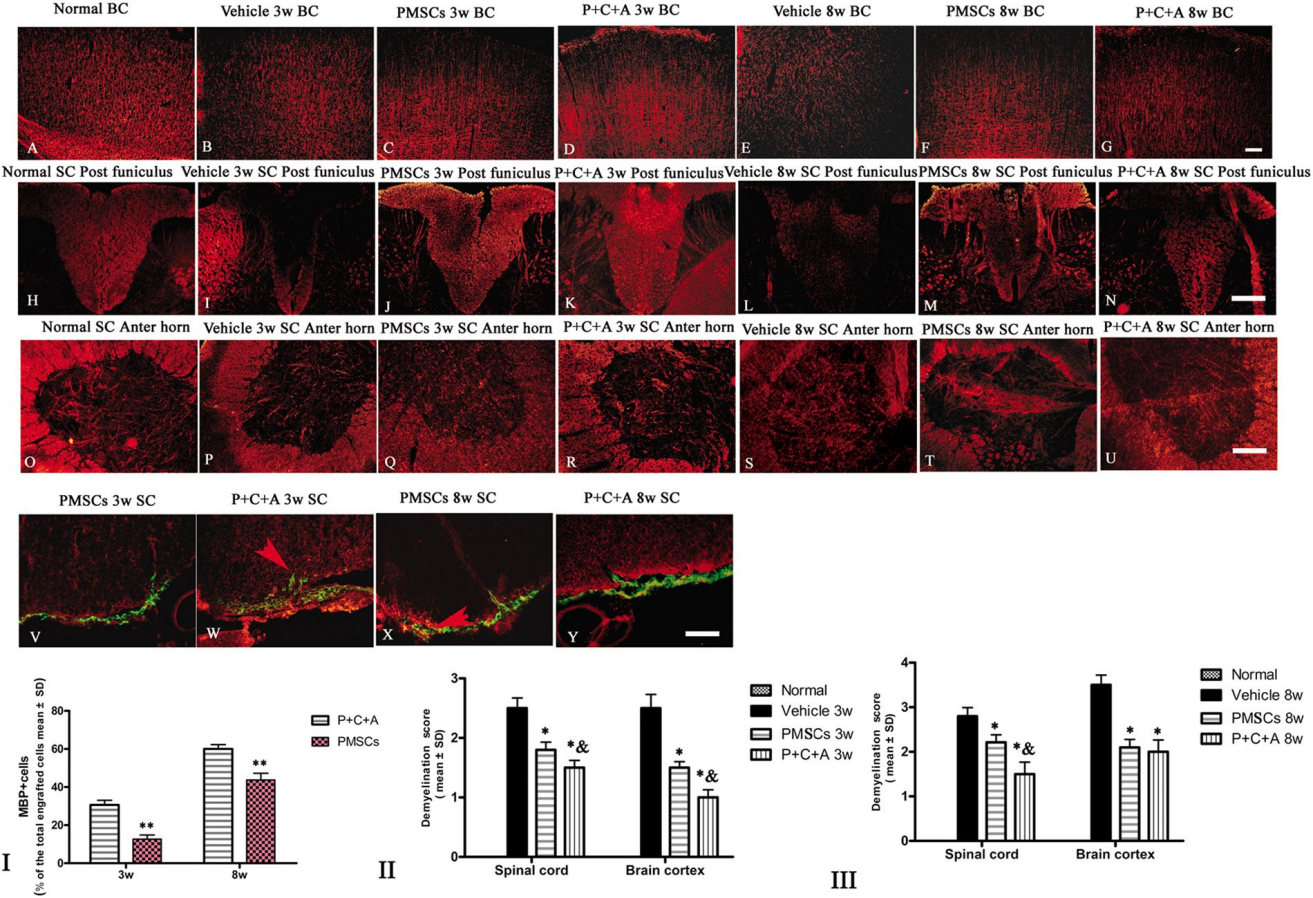
Intravenous C16 and Ang-1 enhanced the efficacy of PMSC therapy for inhibiting demyelination in the EAE rat model. (**A**–**U**) Immunofluorescence staining of brain cortex and spinal cord specimens for MBP (red) at 3 and 8 weeks pi. SC denotes transverse sections through the anterior horn of the lumbar spine, and BC denotes coronal sections of the motor cortex. (**V**–**Y**) Immunofluorescence staining of PMSC grafts (green) for MBP (red) at 3 and 8 weeks pi. Arrows indicate PMSC homing to the parenchyma. Scale bar = 100 μm. (**I**) Percentage of MBP^+^ cells in all engrafted PMSCs at 3 and 8 weeks pi. n = 5, **P < 0.01 vs. P + C + A (at 3 weeks pi, ES = 1.69, *P* < 0.0001; at 8 weeks pi, ES = 0.92, *P* < 0.0001). (**II**,**III**) Demyelination scores in BC and SC at 3 (II) and 8 (III) weeks pi. n = 5, *P < 0.01 vs. vehicle, ^&^*P* < 0.05 vs. PMSCs. (II) In SC, PMSCs vs. vehicle: ES = 15.41, *P* < 0.0001; P + C + A vs. vehicle: E = 22.93, *P* < 0.0001; P + C + A vs. PMSCs: ES = 9.86, *P* = 0.0025. In BC, PMSCs vs. vehicle: ES = 15.96, *P* < 0.0001; P + C + A vs. vehicle: ES = 21.51, *P* < 0.0001; P + C + A vs. PMSCs: ES = 17.23, *P* = 0.0001. (III) In SC, PMSCs vs. vehicle: ES = 9.28, *P* = 0.0004; P + C + A vs. vehicle: ES = 11.99, *P* < 0.0001; P + C + A vs. PMSCs: ES = 7.05, *P* = 0.005. In BC, PMSCs vs. vehicle: ES = 17.39, *P* < 0.0001; P + C + A vs. vehicle: ES = 12.48, *P* < 0.0001; P + C + A vs. PMSCs: ES = 0.95, *P* = 0.26.

EAE is characterized by demyelination, perivascular edema, and neuronal cell death in the CNS. In this study, demyelination was assessed by western blotting and immunofluorescence staining for MBP, a specific marker of myelination. In addition, morphological changes in the myelin sheath, neurons, and blood vessels in the CNS were examined by TEM. Western blotting revealed significantly decreased MBP expression in the brain cortex of rats with EAE at 3 and 8 weeks pi (Fig. 5A–C, *P* < 0.05). Immunofluorescence staining for MBP showed myelin disruption and demyelination in the brain cortex and spinal cords of rats with EAE (Fig. 6B,E,I,L). EAE-associated MBP loss and demyelination were ameliorated by treatment with PMSCs, either alone or in combination with intravenous C16 and Ang-1 (Fig. 6A–U, I–III; *P* < 0.05), with the P + C + A group showing more effective prevention of MBP loss and demyelination than PMSC treatment alone (Fig. 6I-III, *P* < 0.05). Engrafted PMSCs (green) were detected in the subarachnoid space, with infiltration into the parenchyma (Fig. 6V–Y). MBP, a marker of the oligodendrocyte lineage, was detected in the engrafted PMSCs in both the PMSCs and P + C + A groups, with relatively higher levels detected in the P + C + A group (Fig. 6V–Y, I; *P* < 0.05). LFB staining for myelin revealed loss of myelin where inflammatory cell infiltration was observed in the brain cortex and spinal cords of rats with EAE, and these EAE-associated changes were reversed by treatment with PMSCs, alone or in combination with intravenous C16 and Ang-1 (Fig. S6).

**Figure 7:**
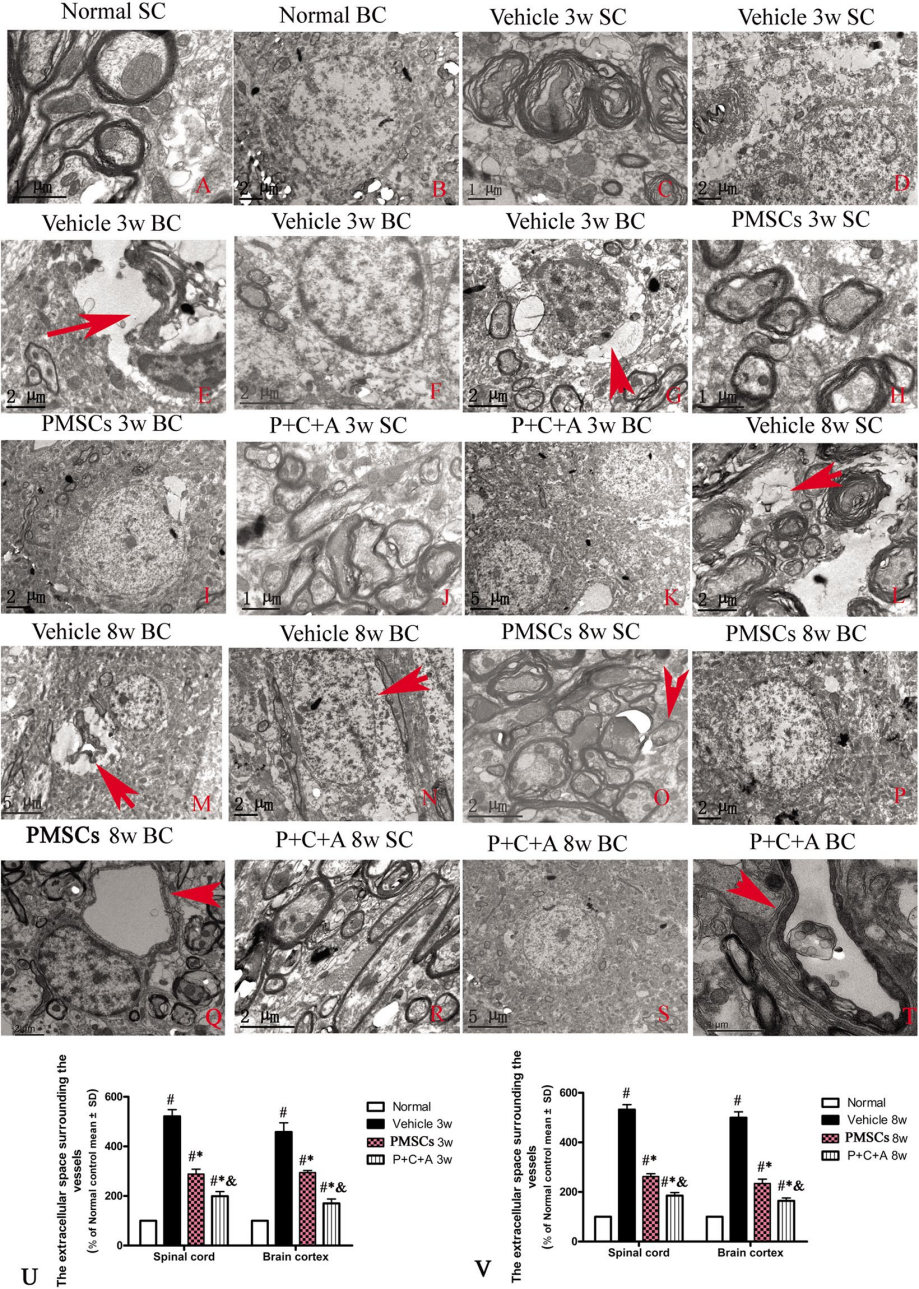
Electron micrographs demonstrating the prevention of perivascular edema, demyelination/axon loss, and neuronal apoptosis/necrosis in the rat EAE model by PMSC and P + C + A treatment. (**A**,**B**) Normal control rats. (**A**) normal myelinated axons exhibiting dark, ring-shaped myelin sheaths surrounding axons, (**B**) normal neuronal nuclei with uncondensed chromatin. (**C**–**G**) Vehicle-treated EAE rats at 3 weeks pi. (**C**) myelin sheath displaying splitting, vacuoles, loose and fused changes, and shrunken, atrophied axons, (**D**,**E**) tissue edema (**D**) and severe blood vessel leakage (**E**, arrow) detected in the extracellular space surrounding the vessels, (**F**) a neuron showing signs of apoptosis with a shrunken nucleus and condensed, fragmented, and marginated nuclear chromatin, (**G**) an extravasated inflammatory cell in tissue edema. (**H**–**K**) PMSC- (**H**,**I**) and P + C + A- (**J**,**K**) treated EAE rats at 3 weeks pi. Myelin sheath splitting, axonal loss, and perivascular edema were reduced, and neuron nuclei displayed relatively normal morphology. (**L**–**N**) Vehicle-treated EAE rats at 8 weeks pi. Many myelin lamellae were still undergoing vesicular disintegration and demyelination (**L**), with some fibers being completely lost or only showing an empty circle of remaining myelin (arrow in **L**). Perivascular edema and leakage as well as extravasated inflammatory cells were still present (**M**). Some neurons exhibited signs of necrosis such as large vacuoles and degenerated organelles in the perikaryon, ruptured cytoplasmic membranes, and oncolytic chromatins (arrow in **N**). (**O**–**T**) PMSC- (**O–Q**) and P + C + A- (**R**–**T**) treated EAE rats at 8 weeks pi. Newly formed myelin sheaths were detected surrounding intact axons (arrow in **O**). The morphology of neuron nuclei became relatively normal, especially in the P + C + A-treated group. Perivascular edema and leakage were evidently alleviated. K, M, S, scale bar = 5 µm; **B,D,E,F,G,I,L,N,O–R**, scale bar = 2 µm; **A,C,H,J,T**, scale bar = 1 µm. (SC) Transverse sections through the anterior horn of the lumbar spinal. (BC) Coronal sections of the motor cortex. (**U,V**) The calculations of extracellular space surrounding the vessels at 3 (**U**) and 8 (**V**) weeks pi. n = 5, ^#^*P* < 0.05 vs. normal, ^*^P < 0.05 vs. vehicle, ^&^*P* < 0.05 vs. PMSCs. (**U**) In SC, PMSCs vs. vehicle: ES = 0.2, *P* < 0.0001; P + C + A vs. vehicle: ES = 0.3, *P* < 0.0001; P + C + A vs. PMSCs: ES = 0.12, *P* < 0.0001. In BC, PMSCs vs. vehicle: ES = 0.11, *P* = 0.0003; P + C + A vs. vehicle: ES = 0.17, *P* < 0.0001; P + C + A vs. PMSCs: ES = 0.32, *P* < 0.0001. (**V**) In SC, PMSCs vs. vehicle: ES = 0.51, *P* < 0.0001; P + C + A vs. vehicle: ES = 0.63, *P* < 0.0001; P + C + A vs. PMSCs: ES = 0.27, *P* < 0.0001. In BC, PMSCs vs. vehicle: ES = 0.31, *P* < 0.0001; P + C + A vs. vehicle: ES = 0.49, *P* < 0.0001; P + C + A vs. PMSCs: ES = 0.17, *P* < 0.0001.

TEM images of the brain cortex and spinal cord of normal rats showed neurons with an intact myelin sheath as well as well-defined nuclei (Fig. 7A,B). However, neurons in rats with EAE began to display noticeable myelin sheath splitting as well as vacuolar changes by 3 weeks pi (Fig. 7C). At 8 weeks pi, myelin lamellae disintegration had worsened, with partial or complete loss of the nerve fibers (Fig. 7L; arrow points to loss of nerve fiber). Neuronal apoptosis (Fig. 7F) and inflammatory cell infiltration into the parenchyma (Fig. 7G; arrow indicates an extravasated inflammatory cell in edema tissue) were detected at 3 weeks pi. Some neurons displayed morphological signs of necrosis, including the presence of large vacuoles, degenerated organelles, ruptured cytoplasmic membranes, and oncolytic chromatin (Fig. 7N; arrow indicates oncolytic chromatin). Perivascular edema was observed at 3 weeks pi and worsened over time (Fig. 7D,E,M; arrows in E and M indicate perivascular edema). The reduced extracellular space surrounding the vessels at 3 and 8 weeks pi in PMSCs-treated rats, especially those co-treated with C16 and Ang-1, indicated alleviation of perivascular edema (Fig. 7Q,T,U,V; *P* < 0.05). Moreover, treatment with PMSCs, either alone or in combination with C16 and Ang-1, effectively prevented myelin sheath splitting (Fig. 7H,J) and neuronal apoptosis at 3 weeks pi (Fig. 7I,K). At 8 weeks pi, rats in the PMSCs and P + C + A groups displayed only mild demyelination along with formation of new myelin sheaths around intact axons (Fig. 7O–T; arrow in O indicates newly formed myelin sheath). The morphological changes in subcellular organelles and nuclei were also largely reversed, especially in the P + C + A group (Fig. 7P–T).

### Intravenous C16 and Ang-1 enhanced the efficacy of PMSCs for preventing axonal loss in rats with EAE

**Figure 8:**
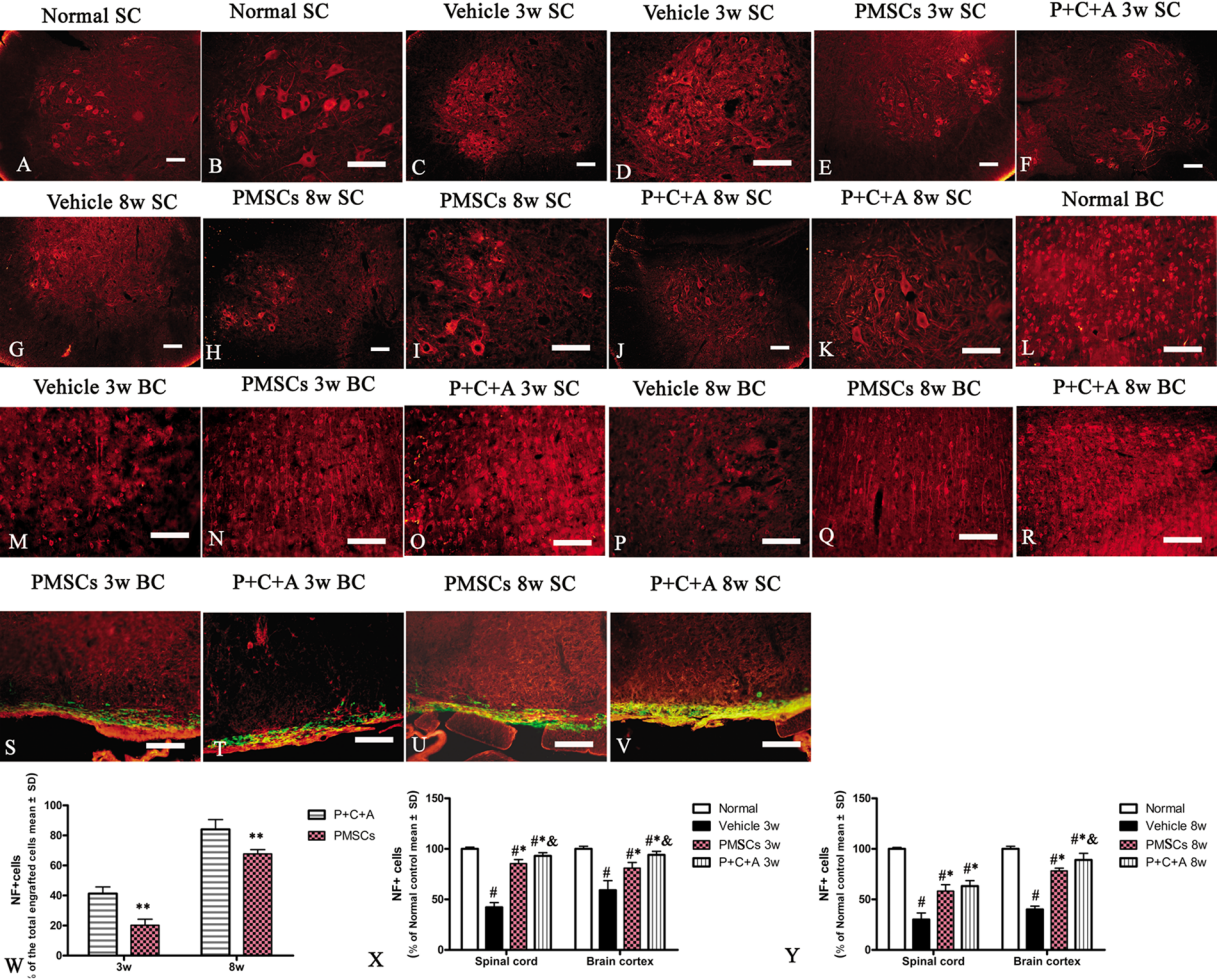
Intravenous C16 and Ang-1 enhanced the efficacy of PMSC therapy for preventing axonal loss in the EAE rat model. (**A**–**R**) Immunofluorescence staining of brain cortex and spinal cord specimens for NF-200 (red) at 3 and 8 weeks pi. SC denotes transverse sections through the anterior horn of the lumbar spine, and BC denotes coronal sections of the motor cortex. (**S**–**V**) Immunofluorescence staining of PMSC grafts (green) for NF-200 (red) at 3 and 8 weeks pi. Scale bars, 100 μm. (**W**) Percentage of NF-200^+^ cells in all engrafted PMSCs at 3 and 8 weeks pi. n = 6, **P < 0.01 vs. PMSCs (at 3 weeks pi: ES = 0.59, *P* < 0.0001; at 8 weeks pi: ES = 0.32, *P* = 0.000443). (**X**,**Y**) Relative NF-200^+^ cell counts in BC and SC at 3 (**X**) and 8 (**Y**) weeks pi. n = 6, ^#^*P* < 0.05 vs. normal, ^*^P < 0.05 vs. vehicle, ^&^*P* < 0.05 vs. PMSCs. (**X**) In SC, PMSCs vs. vehicle: ES = 1.15, *P* < 0.0001; P + C + A vs. vehicle: ES = 1.64, *P* < 0.0001; P + C + A vs. PMSCs: ES = 0.3, *P* = 0.005. In BC, PMSCs vs. vehicle: ES = 0.18, *P* = 0.001; P + C + A vs. vehicle: ES = 0.34, *P* = 0.0003; P + C + A vs. PMSCs: ES = 0.18, *P* = 0.001. (Y) In SC, PMSCs vs. vehicle: ES = 0.34, *P* < 0.0001; P + C + A vs. vehicle: ES = 0.47, *P* < 0.0001; P + C + A vs. PMSCs: ES = 0.07, *P* = 0.11. In BC, PMSCs vs. vehicle: ES = 2.14, *P* < 0.0001; P + C + A vs. vehicle: ES = 0.93, *P* < 0.0001; P + C + A vs. PMSCs: ES = 0.22, *P* = 0.005.

Changes in the morphology of axons were examined by immunofluorescence staining for NF-200. Axonal degeneration was assessed by Bielschowsky’s silver staining as well as western blot analysis of NF-200 expression. Immunofluorescence staining for NF-200 and Bielschowsky’s silver staining revealed reduced axonal density as well as degeneration of the remaining axons in rats with EAE at 3 weeks pi, which worsened over time (Fig. 8B,E,I,L). Treatment with PMSCs, alone (Fig. 8C,F,J,M; Fig. S7C,G,J,M) or in combination with intravenous C16 and Ang-1 (Fig. 8D,G,K,N; Fig. S7D,H,K,N), restored axonal density and morphology, with the P + C + A treatment showing better efficacy than PMSC therapy alone (Fig. S7O,P; *P* < 0.05). Moreover, western blot and RT-PCR analysis revealed diminished NF-200 levels in the brain cortex of rats with EAE, and treatment with PMSCs, alone or in combination with intravenous C16 and Ang-1, ameliorated NF-200 loss, with the P + C + A treatment showing a relatively more prominent effect at later stage of clinic process (Fig. 5D–F, Fig. SP3C,D: *P* < 0.05). NF-200 was detected in the engrafted PMSCs in both PMSCs and P + C + A groups (Fig. 8S–V), with the P + C + A group showing a higher percentage of NF-200-positive cells (Fig. 8W, P < 0.05).

### Intravenous C16 and Ang-1 upregulated neurotropic protein expression and enhanced the efficacy of PMSC therapy for preventing neuronal apoptosis in rats with EAE

**Figure 9:**
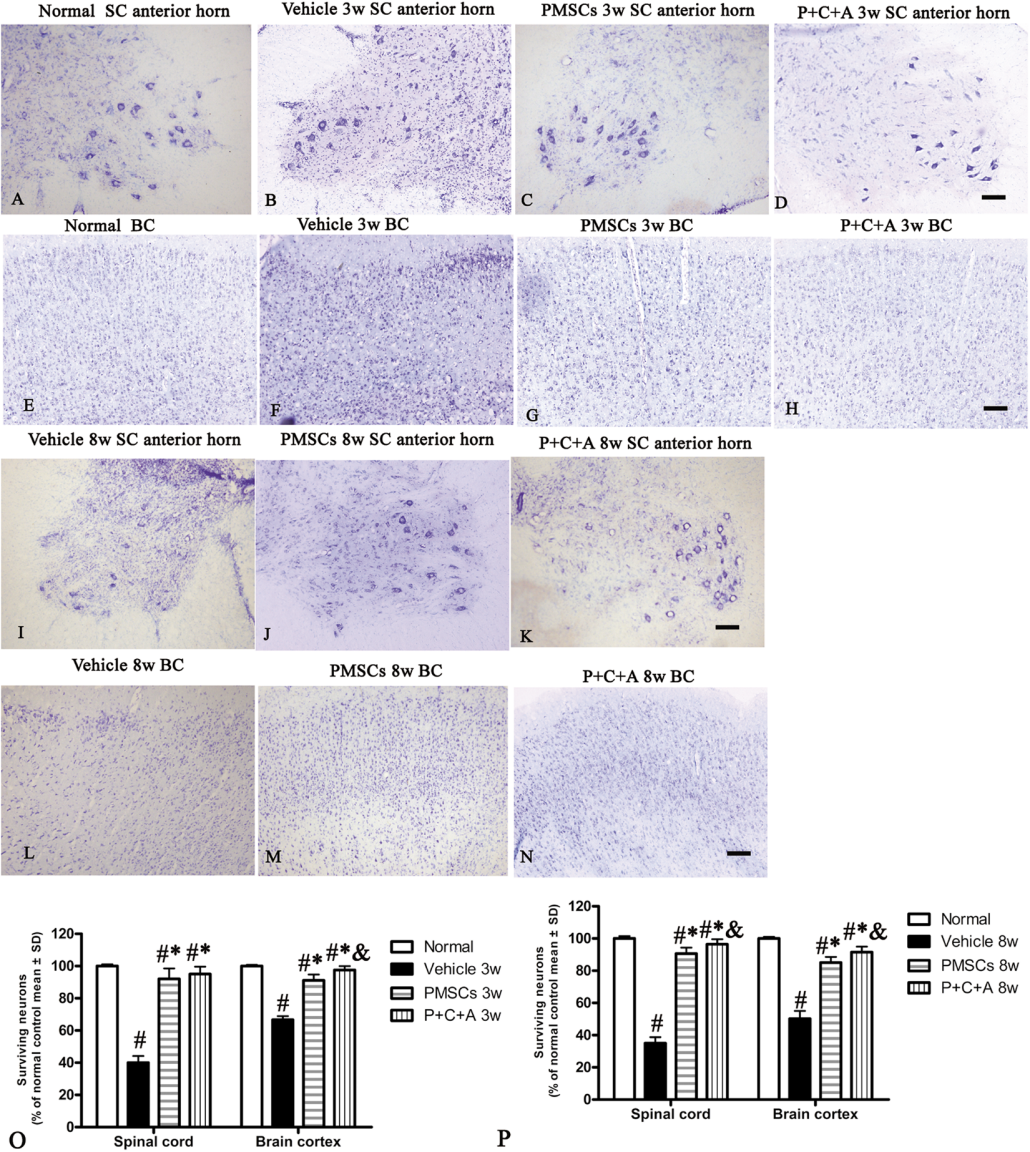
Intravenous C16 and Ang-1 enhanced the efficacy of PMSC therapy for preventing neuronal loss in the EAE rat model. (**A**–**N**) Nissl staining of brain cortex and spinal cord specimens at 3 and 8 weeks pi. SC denotes transverse sections through the anterior horn of the lumbar spine, and BC denotes coronal sections of the motor cortex. Scale bars, 100 µm. (**O**,**P**) Relative numbers of surviving neurons at 3 (**O**) and 8 (**P**) weeks pi. n = 5, ^#^*P* < 0.05 vs. normal, ^*^P < 0.05 vs. vehicle, ^&^*P* < 0.05 vs. PMSCs. (**O**) In SC, PMSCs vs. vehicle: ES = 0.87, *P* < 0.0001; P + C + A vs. vehicle: ES = 1.42, *P* < 0.0001; P + C + A vs. PMSCs: ES = 0.05, *P* = 0.21. In BC, PMSCs vs. vehicle: ES = 1.37, *P* < 0.0001; P + C + A vs. vehicle: ES = 2.94, *P* < 0.0001; P + C + A vs. PMSCs: ES = 0.36, *P* = 0.004. (**P**) In SC, PMSCs vs. vehicle: ES = 2.08, *P* < 0.0001; P + C + A vs. vehicle: ES = 2.72, *P* < 0.0001, P + C + A vs. PMSCs: ES = 0.28, *P* = 0.009. In BC, PMSCs vs. vehicle: ES = 0.97, *P* < 0.0001; P + C + A vs. vehicle: ES = 1.2, *P* < 0.0001; P + C + A vs. PMSCs: ES = 0.28, *P* = 0.01.

BDNF supports neuronal differentiation, growth, and survival^22,23^, and GAP-43 is a crucial component of the axon and presynaptic terminal^24,25^. p75NTR is implicated in the regulation of both synaptic transmission and axonal elongation^26^. To further elucidate the molecular mechanisms underlying the neuroprotective effects of PMSCs, we examined the expression of BDNF, p75NTR, and GAP-43 as well as expression of the apoptosis marker caspase-3 by western blotting, RT-PCR and immunofluorescence staining. The results revealed increased GAP-43 and p75NTR but decreased BDNF levels in the CNS of rats with EAE (Fig. 5G–O; Fig. S3E–J, Fig. S8–10). Treatment with PMSCs, alone or in combination with intravenous C16 and Ang-1, significantly upregulated GAP-43, p75NTR and BDNF expression, with relatively more prominent effects detected in the P + C + A group (Figs 5G–O, S3E–J; S8–10; *P* < 0.05). Western blotting revealed drastically increased caspase-3 levels in the brain cortex of rats with EAE (Fig. 5P–R), and immunofluorescence staining revealed elevated caspase-3 expression in the multipolar motor neurons of the spinal cord anterior horn as well as the pyramid-shaped motor neurons of the precentral gyrus (Fig. S11). Treatment with PMSCs, either alone or in combination with intravenous C16 and Ang-1, inhibited caspase-3 upregulation in rats with EAE, with the P + C + A treatment showing a more potent effect (Fig. 5P–R, S11; *P* < 0.05). Nissl staining showed visible neuronal loss in the CNS of rats with EAE that progressed over time (Fig. 9A–N). In the PMSCs and P + C + A groups, the numbers of surviving neurons in the brain cortex and spinal cord were increased (Fig. 9A–N), with the P + C + A treatment showing a relatively more potent neuronal cell-preservation effect (Fig. 9O,P; *P* < 0.05).

### Intravenous C16 and Ang-1 upregulated the expression of the neuronal–glial lineage markers in engrafted PMSCs

**Figure 10:**
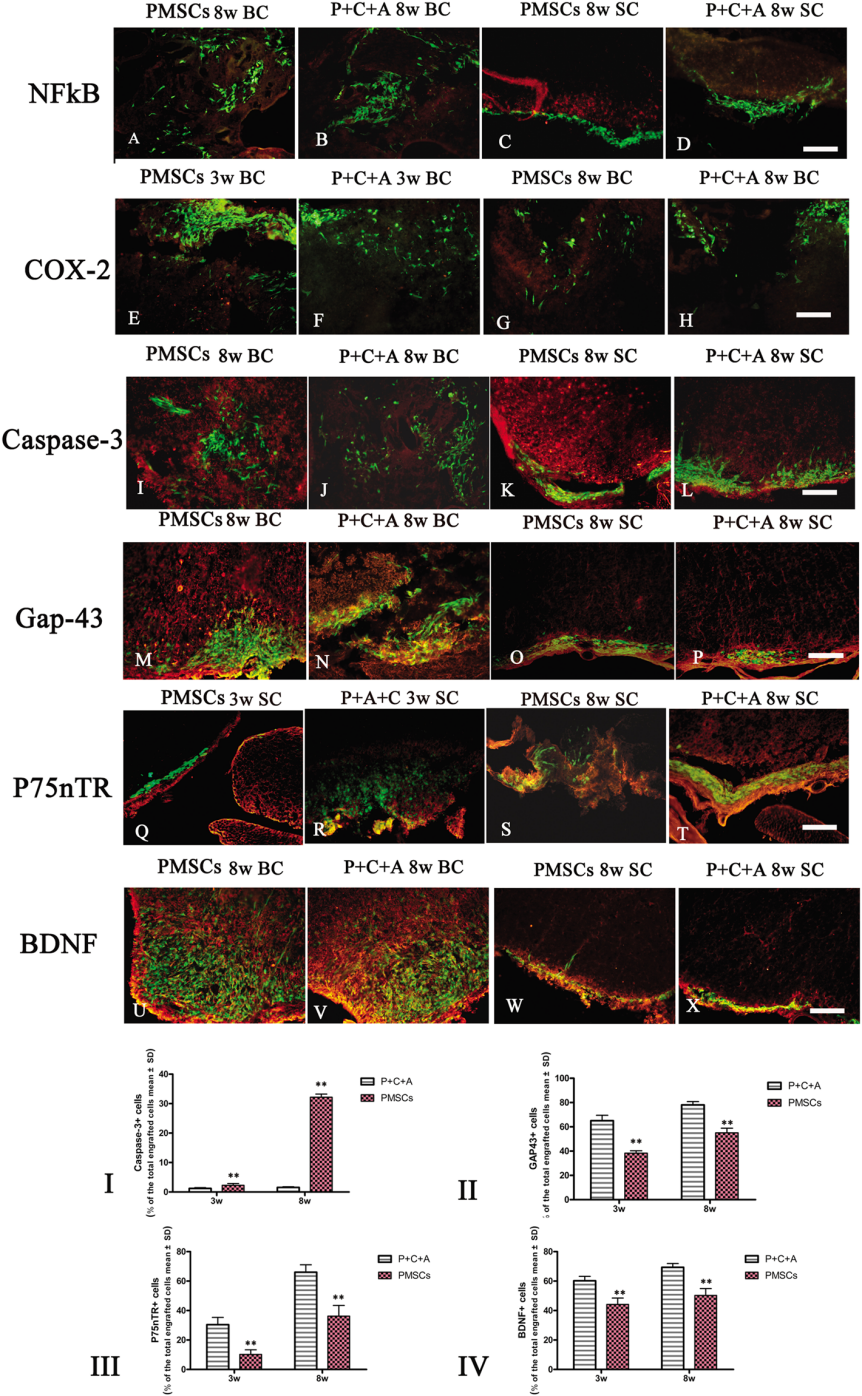
Immunofluorescence staining of PMSC grafts (green) in brain cortex and spinal cord specimens for NF-κB (red, **A**–**D**), COX-2 (red, **E**–**H**), caspase-3 (red, **I**–**L**), GAP-43 (red, **M**–**P**), p75NTR (red, **Q**–**T**), and BDNF (red, **U**–**X**) at 3 and 8 weeks pi. SC denotes transverse sections through the anterior horn of the lumbar spine, and BC denotes coronal sections of the motor cortex. Scale bars, 100 μm. (**I**–**IV**) Percentages of cells stained positively for caspase-3 (I), GAP-43 (II), p75NTR (III), and BDNF (IV) among all engrafted PMSCs. n = 5, **P < 0.01 vs. P + C + A. (I) ES = 2.63, *P* = 0.0003 at 3 weeks pi; ES = 24.77, *P* < 0.0001 at 8 weeks pi. (II) ES = 1.09, *P* < 0.0001 at 3 weeks pi; ES = 1.05, *P* < 0.0001 at 8 weeks pi. (III) ES = 0.6, *P* < 0.0001 at 3 weeks pi; ES = 0.37, *P* < 0.0001 at 8 weeks pi. (IV) ES = 0.57, *P* < 0.0001 at 3 weeks pi; ES = 0.67, *P* < 0.0001 at 8 weeks pi.

We detected expression of the neuronal-glial lineage markers NF-200 and MBP (Figs 6V–Y and 8O–R) but not the astrocyte marker GFAP (Fig. 4O–R) in PMSCs homing to the CNS of rats with EAE. Intravenous C16 and Ang-1 increased the expression of NF-200 and MBP in these cells (Figs 6I and 8S), suggesting that the combinatorial treatment can promote differentiation of engrafted PMSCs along neuronal–glial lineages. We next examined the expression of the neurotropic proteins GAP-43, p75NTR, and BDNF; the proinflammatory factors NF-κB and COX-2; and the apoptosis marker caspase-3 in engrafted PMSCs by immunofluorescence staining. NF-κB and COX-2 were not detected (Fig. 10A,C,E,G), but caspase-3 was detected at low levels at 8 weeks pi (Fig. 10I,K). GAP-43, p75NTR, and BDNF were all detected in the engrafted PMSCs (Fig. 10M,O,Q,S,U,W). Intravenous C16 and Ang-1 upregulated GAP-43, p75NTR, and BDNF (Fig. 10II–IV, *P* < 0.05) and downregulated caspase-3 (Fig. 10I, *P* < 0.05) in the engrafted cells.

## Discussion

In a recent side-by-side comparison of PMSC versus EMSC therapy for EAE, PMSCs demonstrated similar efficacy to EMSCs at inhibiting inflammatory cell infiltration, demyelination, axonal loss, and neurological dysfunction^12^. Moreover, both PMSCs and EMSCs demonstrated the ability to migrate into the inflamed CNS tissues as well as the potential to differentiate along the neural–glial lineage^12^. These findings supported PMSCs as an ideal cell-based therapy for MS. However, significant inflammatory cell infiltration and inflammation of the CNS remained after PMSC transplantation, which limited the therapeutic potential of this treatment modality^12^.

C16 and Ang-1 have been reported to block inflammatory cell migration by promoting vascular integrity and interfering with leukocyte–endothelial interactions, respectively^13–15^. In addition, because these two molecules act through different mechanisms, they have been shown to work synergistically to mitigate inflammatory responses in EAE^14^. In the present study, intravenous administration of C16 and Ang-1 significantly increased the efficacy of PMSC therapy for reducing CNS inflammation, neuronal injury, and neurological dysfunction in a rat model of EAE.

Similar to our previous findings^12^, transplantation of PMSCs downregulated the proinflammatory molecules TNF-α, IFN-γ, IL-17, NF-κB, and COX-2 and upregulated the anti-inflammatory cytokine TGF-β. The perivascular/parenchymal infiltration of CD68^+^ microglia/macrophages was also attenuated by treatment with PMSCs. These anti-inflammatory effects of PMSCs were enhanced by intravenous administration of C16 and Ang-1.

Our mechanistic studies suggested that C16 and Ang-1 enhanced the efficacy of PMSC therapy by promoting homing of these cells to the inflamed tissues in the CNS as well as enhancing their differentiation/regenerative capacity. Reactive astrogliosis in rats with EAE, which obstructed PMSC transmigration, was attenuated by combinatorial treatment with C16 and Ang-1, leading to more effective PMSC engraftment in the CNS. Transplanted stem cells can produce neurotrophins to stimulate neuronal cell growth in animals with EAE. They can also differentiate along the neuronal–glial lineage to replace damaged neurons^27–30^. C16 and Ang-1 boosted the ability of the engrafted PMSCs to differentiate down the neuronal–glial lineage as assessed by the expression of the neuronal–glial markers NF-200 and MBP in these cells. Moreover, C16 and Ang-1 upregulated BDNF (a key player in neuron survival and differentiation^22,23^), GAP-43 (a driving factor of axonal sprouting and restoration^24,25^), and p75NTR (a regulator of neuron proliferation and maturation^31,32^) and downregulated the apoptosis marker caspase-3 in the engrafted PMSCs. Invasion of inflammatory cells into MSC grafts is considered to negatively impact the survival and function of the grafted cells^12^. In this study, we detected infiltration of CD68^+^ microglia/macrophages into the PMSC grafts in the CNS, mainly localized in the central part of the cellular mass as described in our previous reports^12^. Combinatorial treatment with the C16 and Ang-1 markedly reduced inflammatory cell infiltration into the grafts, which, we believe, contributed to the improved retention, differentiation, and neuroprotective function of the transplanted PMSCs.

The injection of antigens into the anterior chamber of the eye induces a systemic suppression of cell-mediated and humoral immune responses to the antigen. This so called “anterior chamber associated-immune deviation (ACAID)” is largely mediated by TGF-β2 in the aqueous humor acting on ocular antigen-presenting cells (APCs), eventually leading to activation of antigen-specific T regulatory cells (Tregs)^33^. Previous studies have shown that intravenous injection of *in vitro*-generated APCs specific to the encephalitogenic antigens myelin oligodendrocyte glycoprotein (MOG) and/or MBP induces antigen-specific tolerance^33–35^. These encephalitogenic antigen-specific APCs might also augment the efficacy of PMSC and P + C + A therapy for EAE, especially in a MOG/MBP-induced chronic EAE model with pathological features typical of chronic, progressive MS^36^.

Granulocyte macrophage colony-stimulating factor (GM-CSF) has emerged as a putative therapeutic target in MS^37^. Macrophage infiltration is considered a major contributing factor to demyelination in both clinical MS and animal models of EAE. GM-CSF stimulates proliferation and activation of macrophages, monocytes, neutrophils, eosinophils, dendritic cells, and microglia with subsequent induction of pro-inflammatory mediators, and evidence suggests that this cytokine may be involved in the inflammatory processes related to MS^38–40^. Indeed, GM-CSF^−/−^ mice are resistant to EAE and immune cell infiltration in the CNS^37^. However, GM-CSF has also been known to suppress autoimmune diseases such as Crohn’s disease, type-1 diabetes, Myasthenia gravis, and experimental autoimmune thyroiditis by promoting Treg expansion and/or modulating phenotype-specific differentiation of precursor immune cells^41,42^. Thus, GM-CSF could function as a double-edged sword in EAE development. The effects of GM-CSF on PMSC and P + C + A therapy for EAE warrant further investigation.

## Conclusion

Intravenous C16 and Ang-1 increased the efficacy of PMSC therapy for preventing demyelination/neuronal loss and ameliorating neurological dysfunction in a rat model of EAE by inhibiting inflammatory cell infiltration and enhancing PMSC engraftment, survival, differentiation, and neurotrophin production. Further pharmacokinetic and pharmacodynamic studies, preferably in primates, are needed to evaluate the therapeutic potential of this treatment regimen for MS.

## Methods

### Isolation and culture of GFP-expressing rat PMSCs

Green fluorescent protein (GFP)-expressing PMSCs were isolated from rats as previously described^12^. The cells were maintained at 37 °C in a humidified incubator with 5% CO_2_ for 4–5 weeks prior to experimental studies. The medium was exchanged every 3–4 days.

### Animal model of EAE

A total of 77 adult male Lewis rats (9–10 weeks of age, 200–250 g) were obtained from the Center of Laboratory Animal Services at Zhejiang University. The rats were randomly assigned to four groups: normal control group (normal, n = 11), vehicle-treated EAE group (vehicle, n = 22), PMSC-treated EAE group (PMSCs, n = 22), and EAE group treated with PMSCs plus intravenous C16 and Ang-1 (P + C + A, n = 22). EAE was induced as previously described^12^ by subcutaneous injection of guinea pig spinal cord homogenate (GPSCH) emulsified at a 1:1 ratio with complete Freund adjuvant (CFA) containing heat killed *Mycobacterium tuberculosis*. Rats in the normal group received an injection of CFA emulsified at a 1:1 ratio in 0.9% saline. Each rat received an intraperitoneal injection of 300 ng Pertussis toxin (Sigma-Aldrich, St. Louis, MO, USA) in 0.1 ml distilled water immediately after the subcutaneous injection and again 48 h later. Rats in the P + C + A group also received once daily intravenous administration of C16 (2 mg) and Ang-1 (400 µg) until the time of sacrifice, starting immediately after EAE induction. C16 and Ang-1 were purchased from Shanghai Science Peptide Biological Technology Co., Ltd. (Shanghai, China).

The clinical manifestations of EAE were assessed daily until the time of sacrifice. Disease severity was scored on a 5-point scale: 0 = no signs, 1 = partial loss of tail tonicity, 2 = loss of tail tonicity, 3 = unsteady gait and mild paralysis, 4 = hind limb paralysis and incontinence, and 5 = moribund or death^15^. Disease scoring was performed by pathologists blinded to treatment conditions.

All animal studies were approved by the Animal Ethics Committee of Zhejiang University and carried out in accordance with the National Institutes of Health (NIH) Guidelines for the Care and Use of Laboratory Animals.

### PMSC transplantation

One week after EAE induction, rats in the PMSCs and P + C + A groups received intrathecal infusion of 1 × 10^6^ PMSCs into the subarachnoid space as previously described^12^. Rats in the vehicle group received injections of phosphate-buffered saline (PBS) instead.

### Neurophysiological testing

Cortical somatosensory evoked potentials (CSEPs) were recorded as previously described^16–18^ at 3 weeks (peak stage of clinical manifestations) and 8 weeks (recovery stage) pi (n = 5 per group). For measurement of CSEPs, rats were fixed to a stereotaxic frame. A constant current stimulator (Digitimer, Welwyn Garden City, UK) was used to deliver positive current pulses (15 V, 40 ms duration) to produce a maximum SEP (averaged over 30 stimuli). The SEPs from three series of stimulations were amplified, filtered, digitally converted, and stored for post-hoc analysis. Peak positive and negative values were recorded. The results are presented as the mean ± standard deviation (SD) of voltage amplitude (µV) and latency (ms).

Cortical motor evoked potentials (CMEPs) were recorded following previously reported procedures^19,20^ at the same time points (n t 5 per group). Following anesthesia, a midline incision was made on the scalp. The tissues underneath were cleaned, and the cranium exposed. Screw electrodes were implanted to a depth of 0.75 mm over the primary somatomotor cortical areas, gently touching the dura mater. A needle electrode was inserted into the muscle of the hindlimb, and an inactive reference electrode was inserted under the skin, 2 mm from the screw electrode. The somatomotor cortex was stimulated with a train of 10–25 pulses at 10 Hz that evoked visible contralateral hindlimb movement. The signals were recorded and CEMPs were calculated over three independent experiments.

### Tissue collection and processing

Rats in each group were sacrificed at 3 and 8 weeks after immunization (n = 5 per group at each time point). Rats were anesthetized with an intraperitoneal injection of 1% Nembutal (40 mg/kg) and perfused intracardially with cold saline followed by 4% paraformaldehyde in 0.1 M phosphate buffer (PBS, pH 7.4). The spinal cord and brain tissues were carefully dissected. One centimeter of the lumbar spinal cord and half of the brain of each animal were fixed in the same fixative for 4 h and then transferred to 30% sucrose in PBS until the tissue sunk to the bottom of the container. Twenty-micrometer-thick sections were cut on a freezing microtome through the coronal plane of the brain and transverse plane of the spinal cord using a Leica cryostat and then mounted onto 0.02% poly-L-lysine-coated slides. All sections were collected for histological assessment and immunohistological and immunofluorescent staining. The remains of the CNS tissue were fixed in 2.5% glutaraldehyde solution and examined by transmission electron microscopy (TEM).

### Histological assessment

Extravasated macrophages were detected by immunofluorescence staining for CD68, a specific macrophage marker. In digital photomicrographs taken at 200x magnification in three fields per tissue section, the severity of inflammatory cell infiltration was scored from 0–4^43^: 0 = no infiltration, 1 = infiltration only around blood vessels and meninges, 2 = light infiltration in the parenchyma (1–10 cells per section), 3 = moderate infiltration in the parenchyma (11–100 cells per section), and 4 = severe infiltration in the parenchyma (100 + cells per section). Neuronal loss was assessed by Cresyl Violet staining (Nissl staining). Neuronal counts were restricted to cells that displayed a well-defined nucleolus as well as adequate amounts of endoplasmic reticulum.

The severity of axon demyelination was assessed by Luxol fast blue (LFB) staining and immunofluorescence staining for MBP. Demyelination was scored from 0–5^43^: 0 = no demyelination, 1 = rare foci of demyelination, 2 = light demyelination, 3 = confluent perivascular or subpial demyelination, 4 = substantial perivascular and subpial demyelination in at least one half of the spinal cord together with inflammatory cell infiltration in the CNS parenchyma, and 5 = massive perivascular and subpial demyelination across the entire spinal cord along with inflammatory cell infiltration in the CNS parenchyma.

Axonal loss was assessed by Bielschowsky silver staining and scored from 0–3^44^: 0 = no loss, 1 = superficial loss in less than 25% of tissue, 2 = deep loss in over 25% of tissue, and 3 = deep loss encompassing the entire tissue.

### Immunofluorescence staining

Five sections of the brain cortex and anterior horns of the spinal cord from each rat were randomly selected and subjected to immunofluorescence staining. The tissue sections were incubated overnight at 4 °C with anti-NF-200 (1:500; Abcam, Cambridge, MA, USA), anti-GAP-43 (1:100; Santa Cruz Biotechnology, Santa Cruz, CA, USA), anti-activated caspase-3 (1:500; Cayman Chemical, Ann Arbor, MI, USA), anti-BDNF (1:500; Abcam), anti-NF-κB p65 (1:500; Abcam), Anti-p75NTR (1:500; Abcam), anti-MBP (1:500; Abcam), anti*-*glial fibrillary acidic protein (GFAP, 1:200, Thermo Fisher Scientific, Waltham, MA, USA), anti-COX-2 (1:1000; BioVision, Milpitas, CA, USA), and anti-CD68 (1:100; Santa Cruz Biotechnology) antibodies, individually. After washing in PBS, the sections were incubated with TRITC/FITC-conjugated secondary antibodies (1:200; Invitrogen, Carlsbad, CA, USA) for 1 h at 37 °C. The sections were subsequently mounted on glass slides and coverslipped with Antifade Gel Mount Aqueous Mounting Media (Southern Biotech, Birmingham, AL, USA). Stained sections were viewed under a microscope at 200x magnification, and images were taken of three fields per tissue section. Areas stained positively for GFAP, MBP, and CD68 were analyzed using NIH Image software. The numbers of cells stained positively for GAP43, caspase-3, NF-200, BDNF, NF-kB p65, COX-2, Caspase-3, and p75NTR were counted.

### TEM analysis

Sections of the brain cortex and lumbar spinal cord were examined by TEM as described previously^12–15^. Both low and high magnification images were recorded. The extracellular space surrounding the vessels were calculated with NIH Image software.

### Enzyme-linked immunosorbent assay (ELISA)

Peripheral blood samples were collected at 3 and 8 weeks pi (n = 5 per group at each time point). Concentrations of TNF-α, IL-17, and TGF-β were determined using ELISA kits from Abcam. Concentrations of IFN-γ were determined using an ELISA kit from BioLegend Inc. (San Diego, CA, USA). Optical density at 450 nm was recorded, and the data were tabulated using GraphPad Prism 4.0 (GraphPad Software, Inc., San Diego, CA, USA).

### RT-PCR

Rats were sacrificed by decapitation at 3 and 8 weeks pi (n = 5 per group at each time point). Total RNA was extracted from brain tissues using TRIzol reagents (Invitrogen, CA, USA) according to the manufacturer’s instructions. cDNA was synthesized from 2 μl RNA using a cDNA Reverse Transcription Kit (Thermo Fisher Scientific, CA). The expression of NF-200, GAP-43, caspase-3, NF-κB p65, p75NTR, MBP, GFAP, COX-2 and BDNF was determined by PCR amplification followed by agarose gel electrophoresis. The results were normalized to those for GAPDH. All experiments were performed in triplicate.

### Western blotting

Rats were sacrificed by decapitation at 3 and 8 weeks pi (n = 5 per group at each time point). Brain cortex tissues and 10-mm lumbar spinal cord segments were homogenized and analyzed by western blotting as previously described^12–15^.

### Statistical analysis

Data were analyzed using SPSS 13.0 software (SPSS, Inc., Chicago, IL, USA). Differences between groups were identified by two-way analysis of variance (ANOVA) followed by post-hoc Tukey *t*-tests. A P value of <0.05 was considered statistically significant. All statistical graphs were created using GraphPad Prism Version 4.0.

## Electronic supplementary material

Supplemental figures

## References

[1] Conlon P, Oksenberg JR, Zhang J, Steinman L (1999). The immunobiology of multiple sclerosis: an autoimmune disease of the central nervous system. Neurobiol Dis..

[2] Compston A, Coles A (2002). Multiple sclerosis. Lancet..

[3] Payne NL (2013). Distinct immunomodulatory and migratory mechanisms underpin the therapeutic potential of human mesenchymal stem cells in autoimmune demyelination. Cell Transplant..

[4] Cobo M (2013). Mesenchymal stem cells expressing vasoactive intestinal peptide ameliorate symptoms in a model of chronic multiple sclerosis. Cell Transplant..

[5] Payne NL (2012). Early intervention with gene-modified mesenchymal stem cells overexpressing interleukin-4 enhances anti-inflammatory responses and functional recovery in experimental autoimmune demyelination. Cell Adhesion & Migration..

[6] Giuliani M (2011). Long-lasting inhibitory effects of fetal liver mesenchymal stem cells on T-lymphocyte proliferation. PLoS One..

[7] Wang X (2014). Human ESC-derived MSCs outperform bone marrow MSCs in the treatment of an EAE model of multiple sclerosis. Stem Cell Reports..

[8] Drukker M (2006). Human embryonic stem cells and their differentiated derivatives are less susceptible to immune rejection than adult cells. Stem Cells..

[9] Diaz-Prado S (2011). Isolation and characterization of mesenchymal stem cells from human amniotic membrane. Tissue Eng Part C Methods..

[10] Fisher-Shoval Y (2012). Transplantation of placenta-derived mesenchymal stem cells in the EAE mouse model of MS. J Mol Neurosci..

[11] Frohman EM, Racke MK, Raine CS (2006). Multiple sclerosis–the plaque and its pathogenesis. N Engl J Med..

[12] Jiang H, Zhang Y, Tian K, Wang B, Han S (2017). Amelioration of experimental autoimmune encephalomyelitis through transplantation of placental derived mesenchymal stem cells. Sci Rep..

[13] Jiang H, Zhang F, Yang J, Han S (2014). Angiopoietin-1 ameliorates inflammation-induced vascular leakage and improves functional impairment in a rat model of acute experimental autoimmune encephalomyelitis. Exp Neurol..

[14] Wang B, Tian KW, Zhang F, Jiang H, Han S (2016). Angiopoietin-1 and C16 Peptide Attenuate Vascular and Inflammatory Responses in Experimental Allergic Encephalomyelitis. CNS Neurol Disord Drug Targets..

[15] Fang M (2013). C16 peptide shown to prevent leukocyte infiltration and alleviate detrimental inflammation in acute allergic encephalomyelitis model. Neuropharmacology..

[16] All AH (2009). Effect of MOG sensitization on somatosensory evoked potential in Lewis rats. J Neurol Sci..

[17] Troncoso E, Muller D, Czellar S, Zoltan Kiss J (2000). Epicranial sensory evoked potential recordings for repeated assessment of cortical functions in mice. J Neurosci Methods..

[18] Troncoso E (2004). Recovery of evoked potentials, metabolic activity and behavior in a mouse model of somatosensory cortex lesion: role of the neural cell adhesion molecule (NCAM). Cereb Cortex..

[19] Bolay H, Gursoy-Ozdemir Y, Unal I, Dalkara T (2000). Altered mechanisms of motor-evoked potential generation after transient focal cerebral ischemia in the rat: implications for transcranial magnetic stimulation. Brain Res..

[20] Amadio S (2006). Motor evoked potentials in a mouse model of chronic multiple sclerosis. Muscle Nerve..

[21] Brambilla R (2014). Astrocytes play a key role in EAE pathophysiology by orchestrating in the CNS the inflammatory response of resident and peripheral immune cells and by suppressing remyelination. Glia..

[22] Makar TK (2008). Brain derived neurotrophic factor treatment reduces inflammation and apoptosis in experimental allergic encephalomyelitis. J Neurol Sci..

[23] Mariga A, Mitre M, Chao MV (2017). Consequences of brain-derived neurotrophic factor withdrawal in CNS neurons and implications in disease. Neurobiol Dis..

[24] Jacobson RD, Virag I, Skene JH (1986). A protein associated with axon growth, GAP-43, is widely distributed and developmentally regulated in rat CNS. J Neurosci..

[25] Benowitz LI, Routtenberg A (1997). GAP-43: an intrinsic determinant of neuronal development and plasticity. Trends Neurosci..

[26] Dechant G, Barde YA (2002). The neurotrophin receptor p75 (NTR): novel functions and implications for diseases of the nervous system. Nat Neurosci..

[27] Covacu, R. & Brundin, L. Endogenous spinal cord stem cells in multiple sclerosis and its animal model. *J Neuroimmunol* (2016). 10.1016/j.jneuroim.2016.11.006 27884460

[28] Ghasemi N (2014). Transplantation of human adipose-derived stem cells enhances remyelination in lysolecithin-induced focal demyelination of rat spinal cord. Mol Biotechnol..

[29] Yu JW (2016). Synergistic and Superimposed Effect of Bone Marrow-Derived Mesenchymal Stem Cells Combined with Fasudil in Experimental Autoimmune Encephalomyelitis. J Mol Neurosci..

[30] Leite C (2014). Differentiation of human umbilical cord matrix mesenchymal stem cells into neural-like progenitor cells and maturation into an oligodendroglial-like lineage. PLoS One..

[31] Li HY, Zhou XF (2008). Potential conversion of adult clavicle-derived chondrocytes into neural lineage cells *in vitro*. J Cell Physiol..

[32] Wang WX (2011). Nerve growth factor induces cord formation of mesenchymal stem cell by promoting proliferation and activating the PI3K/Akt signaling pathway. Acta Pharmacol Sin..

[33] Farooq SM, Ashour HM (2014). *In vitro*-induced cell-mediated immune deviation to encephalitogenic antigens. Brain Behav Immun..

[34] Farooq SM, Elkhatib WF, Ashour HM (2014). The *in vivo* and *in vitro* induction of anterior chamber associated immune deviation to myelin antigens in C57BL/6 mice. Brain Behav Immun..

[35] Farooq SM, Ashour HM (2013). Eye-mediated induction of specific immune tolerance to encephalitogenic antigens. CNS Neurosci Ther..

[36] Berard JL, Wolak K, Fournier S, David S (2010). Characterization of relapsing-remitting and chronic forms of experimental autoimmune encephalomyelitis in C57BL/6 mice. Glia..

[37] McQualter JL (2001). Granulocyte macrophage colony-stimulating factor: a new putative therapeutic target in multiple sclerosis. J Exp Med..

[38] Croxford AL, Spath S, Becher B (2015). GM-CSF in Neuroinflammation: Licensing Myeloid Cells for Tissue Damage. Trends Immunol..

[39] Reddy PH (2009). Granulocyte-macrophage colony-stimulating factor antibody suppresses microglial activity: implications for anti-inflammatory effects in Alzheimer’s disease and multiple sclerosis. J Neurochem..

[40] Schottelius A (2013). The role of GM-CSF in multiple sclerosis. Drug Res (Stuttg)..

[41] Bhattacharya P (2015). Dual Role of GM-CSF as a Pro-Inflammatory and a Regulatory Cytokine: Implications for Immune Therapy. J Interferon Cytokine Res..

[42] Bhattacharya P (2015). GM-CSF: An immune modulatory cytokine that can suppress autoimmunity. Cytokine..

[43] Ma X (2010). Berberine attenuates experimental autoimmune encephalomyelitis in C57 BL/6 mice. PLoS One..

[44] Yin JX (2010). Centrally administered pertussis toxin inhibits microglia migration to the spinal cord and prevents dissemination of disease in an EAE mouse model. PLoS One..

