# Author Correction: Intravenous C16 and angiopoietin-1 improve the efficacy of placenta-derived mesenchymal stem cell therapy for EAE

**DOI:** 10.1038/s41598-020-65138-2

**Published:** 2020-05-12

**Authors:** Ke-wei Tian, Yuan-yuan Zhang, Hong Jiang, Shu Han

**Affiliations:** 1 Institute of Anatomy and Cell Biology, Medical College, Zhejiang University, 866 Yuhangtang Road, 310058 Hangzhou, China; 2 Department of Electrophysiology, SirRunRunShaw Hospital, Medical College, Zhejiang University, 310016 Hangzhou, Zhejiang Province China

Correction to: *Scientific Reports* 10.1038/s41598-018-22867-9, published online 15 March 2018

This Article contains errors.

In Figure 4, Panel H is a duplication of Panel E. The correct panel 4H is shown below as Figure 1. The quantification was performed based on the correct data.

**Figure 1:**
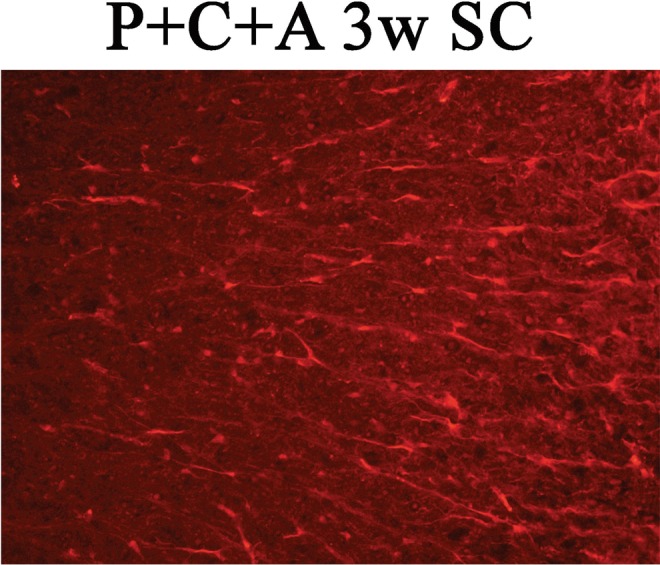
.

This correction does not affect the conclusions of the Article.

