# Supplementary material for: Intravenous C16 and angiopoietin-1 improve the efficacy of placenta-derived mesenchymal stem cell therapy for EAE
Source: Sci Rep. 2018 Mar 15;8:4649. doi: 10.1038/s41598-018-22867-9 (PMC5854598; doi:10.1038/s41598-018-22867-9)
Supplement: Supplementary file 1 — Supplemental figures [file 41598_2018_22867_MOESM1_ESM.pdf]

Intravenous C16 and angiopoietin-1 improve the efficacy of placenta-derived mesenchymal stem cell therapy for EAE

Running title: C16 and Ang-1 improve stem cell therapy for EAE

Ke-wei Tian[1], Yuan-yuan Zhang[1], Hong Jiang[2], Shu Han[1*]

[1]Institute of Anatomy and Cell Biology, Medical College, Zhejiang University, 866 Yuhangtang Road, 310058 Hangzhou, China

[2]Department of Electrophysiology, SirRunRunShaw Hospital, Medical College, Zhejiang University, Hangzhou Zhejiang Province China, 310016

***Corresponding Author: Shu Han**

Institute of Anatomy and Cell Biology, Medical College, Zhejiang University, 866 Yuhangtang Road, 310058 Hangzhou, China

Email: Han00shu@zju.edu.cn; Tel: 0571-88208160; Fax: 0571-88208094

Figure S1

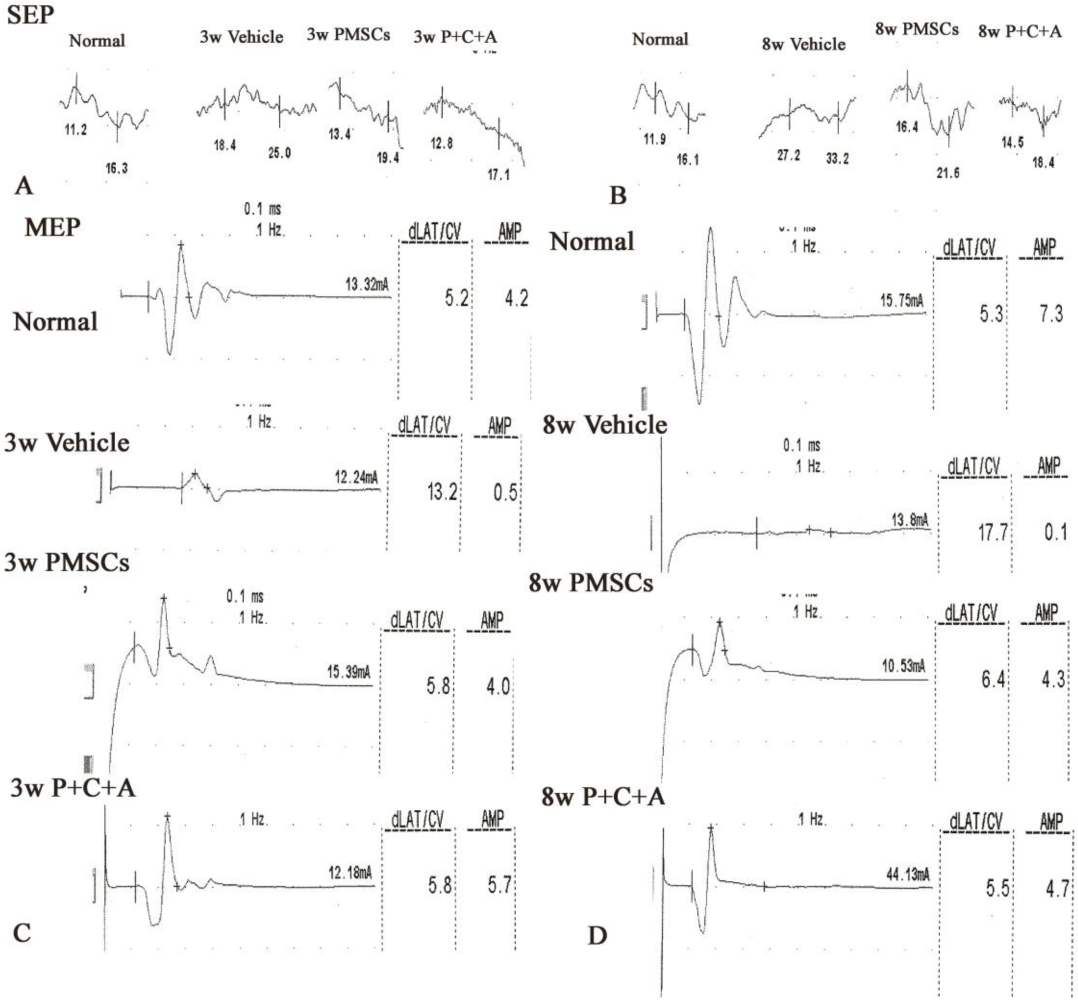
SEP
Normal
3w Vehicle
3w PMSCs
3w P+C+A
11.2
16.3
18.4
25.0
13.4
19.4
12.8
17.1
A
Normal
8w Vehicle
8w PMSCs
8w P+C+A
11.9
16.1
27.2
33.2
16.4
21.6
14.5
18.4
B
MEP
Normal
0.1 ms
1 Hz.
13.32mA
dLAT/CV
AMP
5.2
4.2
3w Vehicle
1 Hz.
12.24mA
13.2
0.5
3w PMSCs
0.1 ms
1 Hz.
15.39mA
5.8
4.0
3w P+C+A
1 Hz.
12.18mA
5.8
5.7
C
Normal
1 Hz.
15.75mA
5.3
7.3
8w Vehicle
0.1 ms
1 Hz.
13.8mA
17.7
0.1
8w PMSCs
1 Hz.
10.53mA
6.4
4.3
8w P+C+A
1 Hz.
44.13mA
5.5
4.7
D

Figure S2

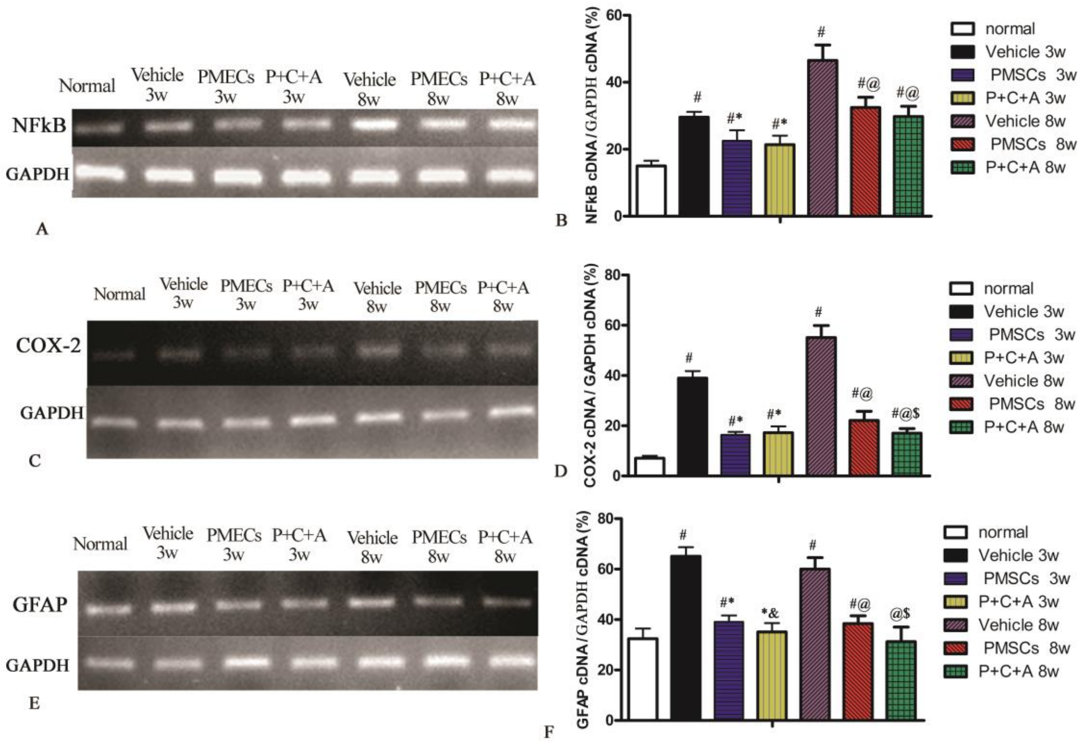
Normal
Vehicle 3w
PMECs 3w
P+C+A 3w
Vehicle 8w
PMECs 8w
P+C+A 8w
NFkB
GAPDH
A
B
NFkB cDNA / GAPDH cDNA (%)
normal
Vehicle 3w
PMSCs 3w
P+C+A 3w
Vehicle 8w
PMSCs 8w
P+C+A 8w
COX-2
GAPDH
C
D
COX-2 cDNA / GAPDH cDNA (%)
GFAP
GAPDH
E
F
GFAP cDNA / GAPDH cDNA (%)

Figure S3

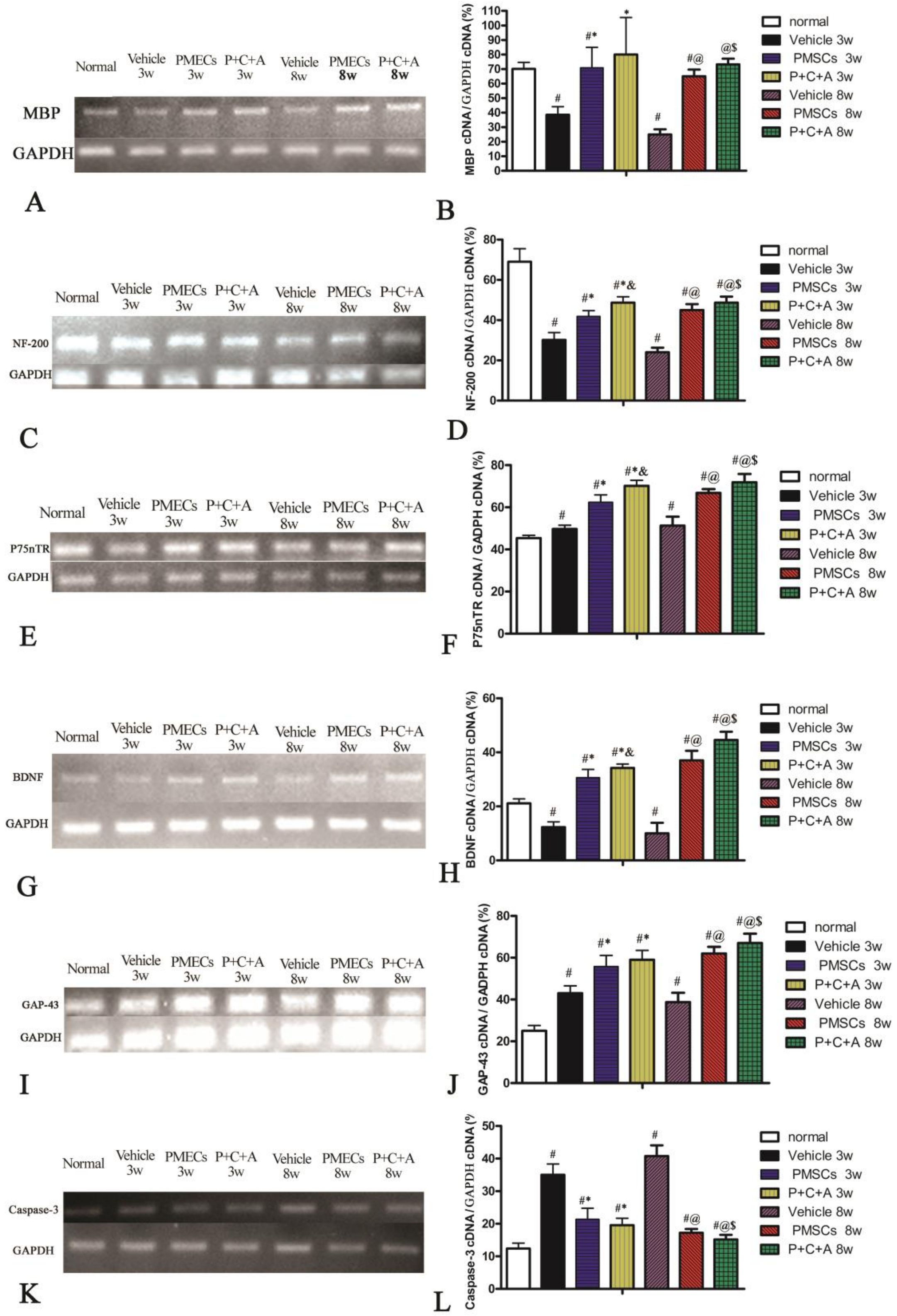
Normal
Vehicle 3w
PMECs 3w
P+C+A 3w
Vehicle 8w
PMECs 8w
P+C+A 8w
MBP
GAPDH
A
MBP cDNA / GAPDH cDNA (%)
normal
Vehicle 3w
PMSCs 3w
P+C+A 3w
Vehicle 8w
PMSCs 8w
P+C+A 8w
B
NF-200
GAPDH
C
NF-200 cDNA / GAPDH cDNA (%)
D
P75nTR
GAPDH
E
P75nTR cDNA / GADPH cDNA (%)
F
BDNF
GAPDH
G
BDNF cDNA / GAPDH cDNA (%)
H
GAP-43
GAPDH
I
GAP-43 cDNA / GADPH cDNA (%)
J
Caspase-3
GAPDH
K
Caspase-3 cDNA / GAPDH cDNA (%)
L

Figure S4

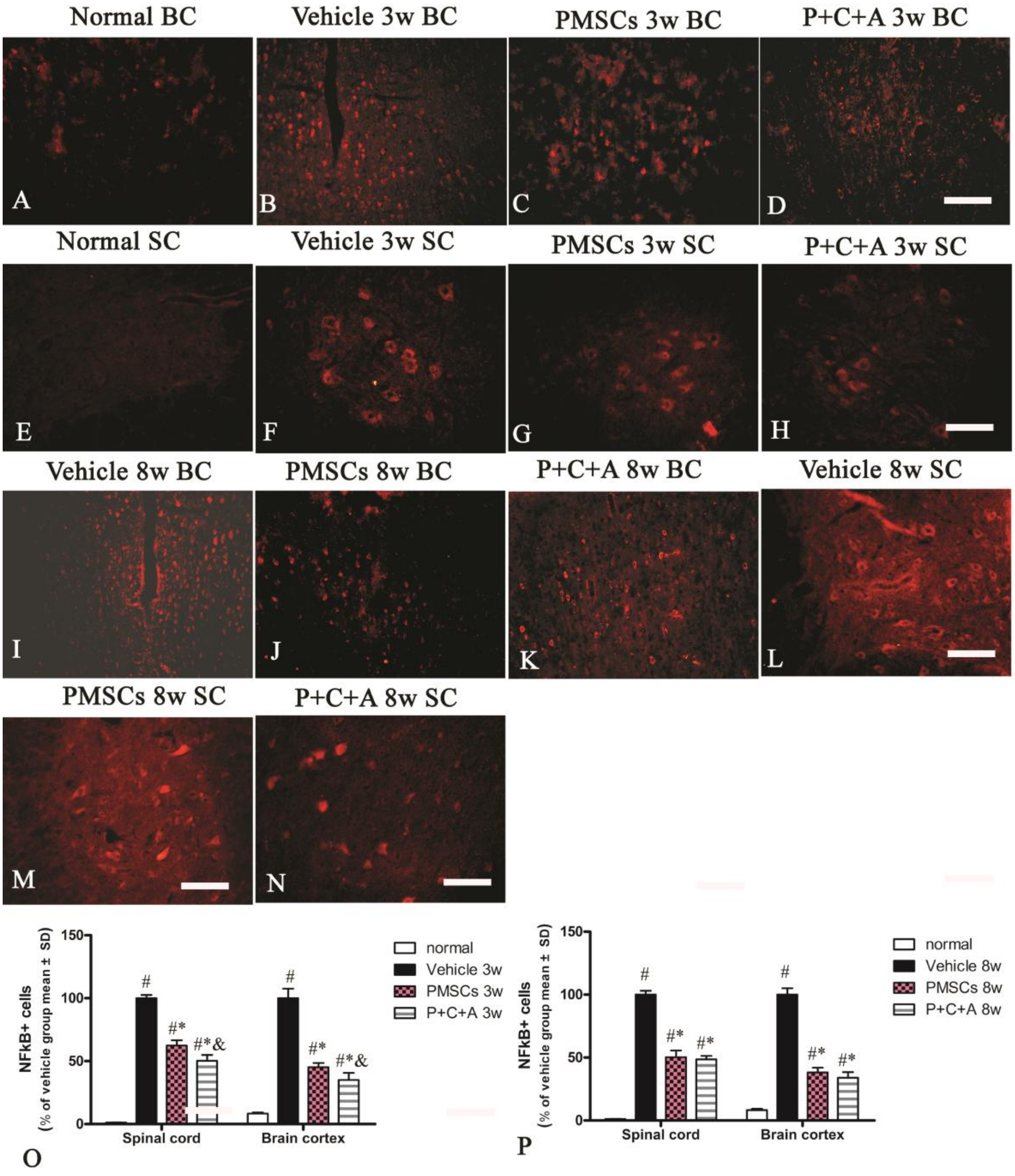

Normal BC
Vehicle 3w BC
PMSCs 3w BC
P+C+A 3w BC
A
B
C
D
Normal SC
Vehicle 3w SC
PMSCs 3w SC
P+C+A 3w SC
E
F
G
H
Vehicle 8w BC
PMSCs 8w BC
P+C+A 8w BC
Vehicle 8w SC
I
J
K
L
PMSCs 8w SC
P+C+A 8w SC
M
N
NFkB+ cells
(% of vehicle group mean ± SD)
150
100
50
0
Spinal cord
Brain cortex
#
#*
#*&
normal
Vehicle 3w
PMSCs 3w
P+C+A 3w
O
normal
Vehicle 8w
PMSCs 8w
P+C+A 8w
P

Figure S5

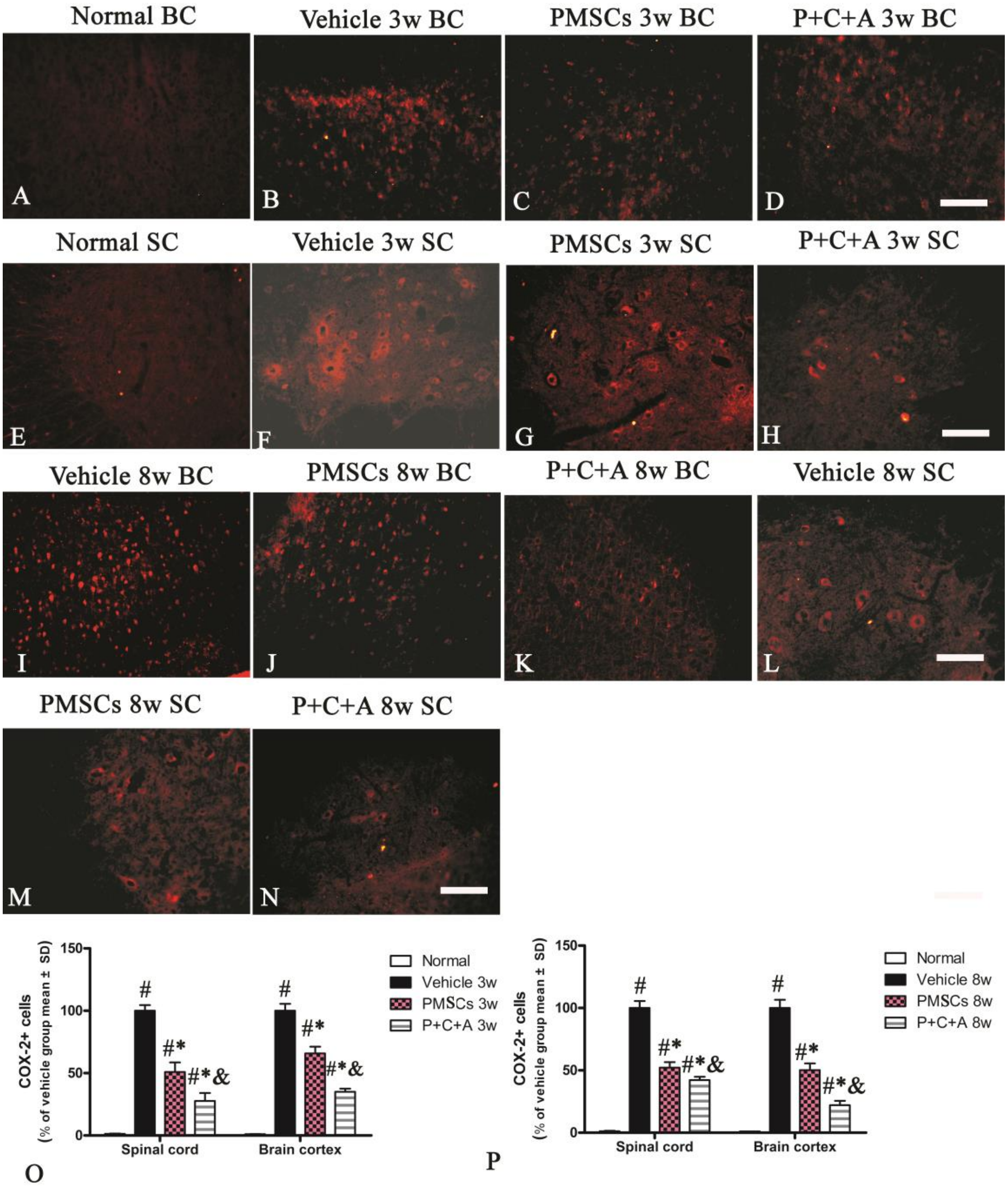
Normal BC
Vehicle 3w BC
PMSCs 3w BC
P+C+A 3w BC
A
B
C
D
Normal SC
Vehicle 3w SC
PMSCs 3w SC
P+C+A 3w SC
E
F
G
H
Vehicle 8w BC
PMSCs 8w BC
P+C+A 8w BC
Vehicle 8w SC
I
J
K
L
PMSCs 8w SC
P+C+A 8w SC
M
N
COX-2+ cells
(% of vehicle group mean ± SD)
150
100
50
0
#
#*
#*&
Spinal cord
Brain cortex
Normal
Vehicle 3w
PMSCs 3w
P+C+A 3w
O
COX-2+ cells
(% of vehicle group mean ± SD)
150
100
50
0
#
#*
#*&
Spinal cord
Brain cortex
Normal
Vehicle 8w
PMSCs 8w
P+C+A 8w
P

Figure S6

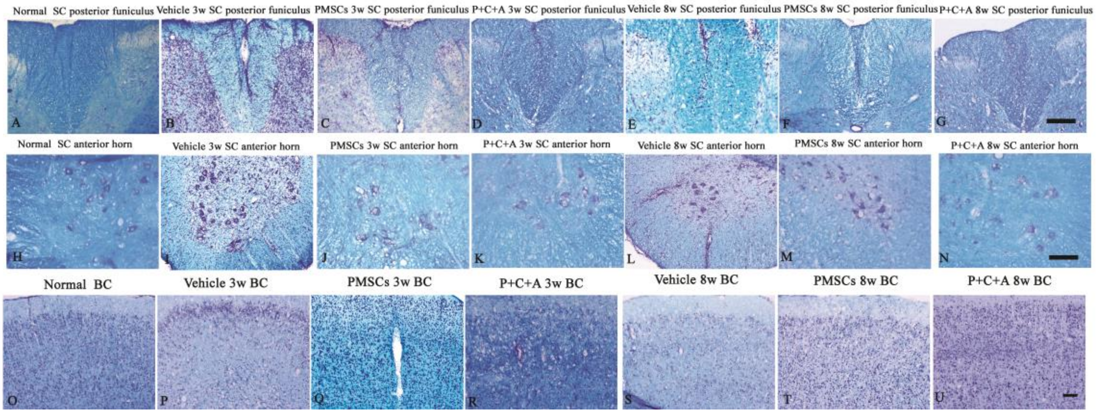

Normal SC posterior funiculus
Vehicle 3w SC posterior funiculus
PMSCs 3w SC posterior funiculus
P+C+A 3w SC posterior funiculus
Vehicle 8w SC posterior funiculus
PMSCs 8w SC posterior funiculus
P+C+A 8w SC posterior funiculus
A
B
C
D
E
F
G
Normal SC anterior horn
Vehicle 3w SC anterior horn
PMSCs 3w SC anterior horn
P+C+A 3w SC anterior horn
Vehicle 8w SC anterior horn
PMSCs 8w SC anterior horn
P+C+A 8w SC anterior horn
H
I
J
K
L
M
N
Normal BC
Vehicle 3w BC
PMSCs 3w BC
P+C+A 3w BC
Vehicle 8w BC
PMSCs 8w BC
P+C+A 8w BC
O
P
Q
R
S
T
U

Figure S7

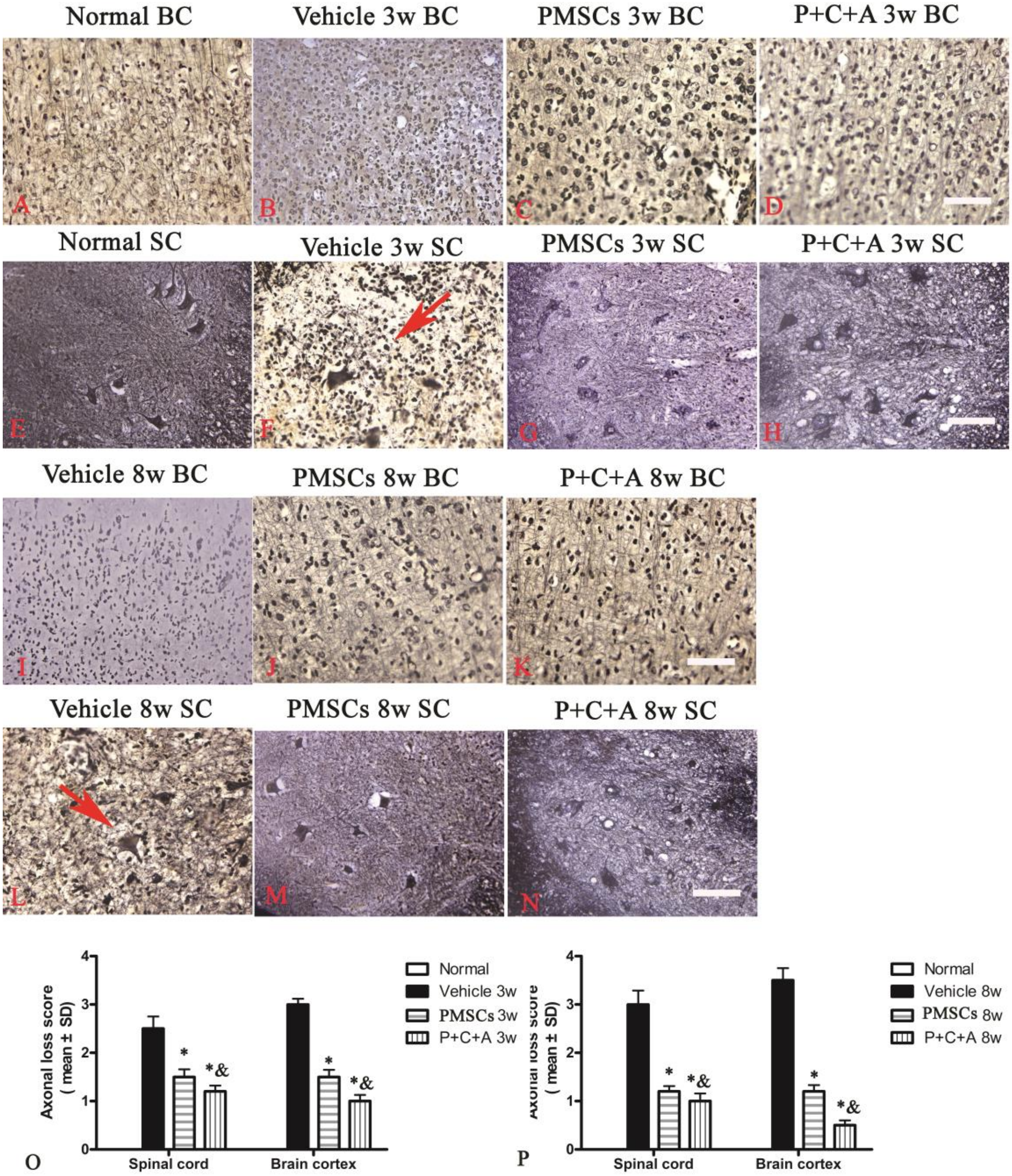

Normal BC
Vehicle 3w BC
PMSCs 3w BC
P+C+A 3w BC
A
B
C
D
Normal SC
Vehicle 3w SC
PMSCs 3w SC
P+C+A 3w SC
E
F
G
H
Vehicle 8w BC
PMSCs 8w BC
P+C+A 8w BC
I
J
K
Vehicle 8w SC
PMSCs 8w SC
P+C+A 8w SC
L
M
N
Axonal loss score (mean ± SD)
Spinal cord
Brain cortex
Normal
Vehicle 3w
PMSCs 3w
P+C+A 3w
*
*&
O
Normal
Vehicle 8w
PMSCs 8w
P+C+A 8w
P

Figure S8

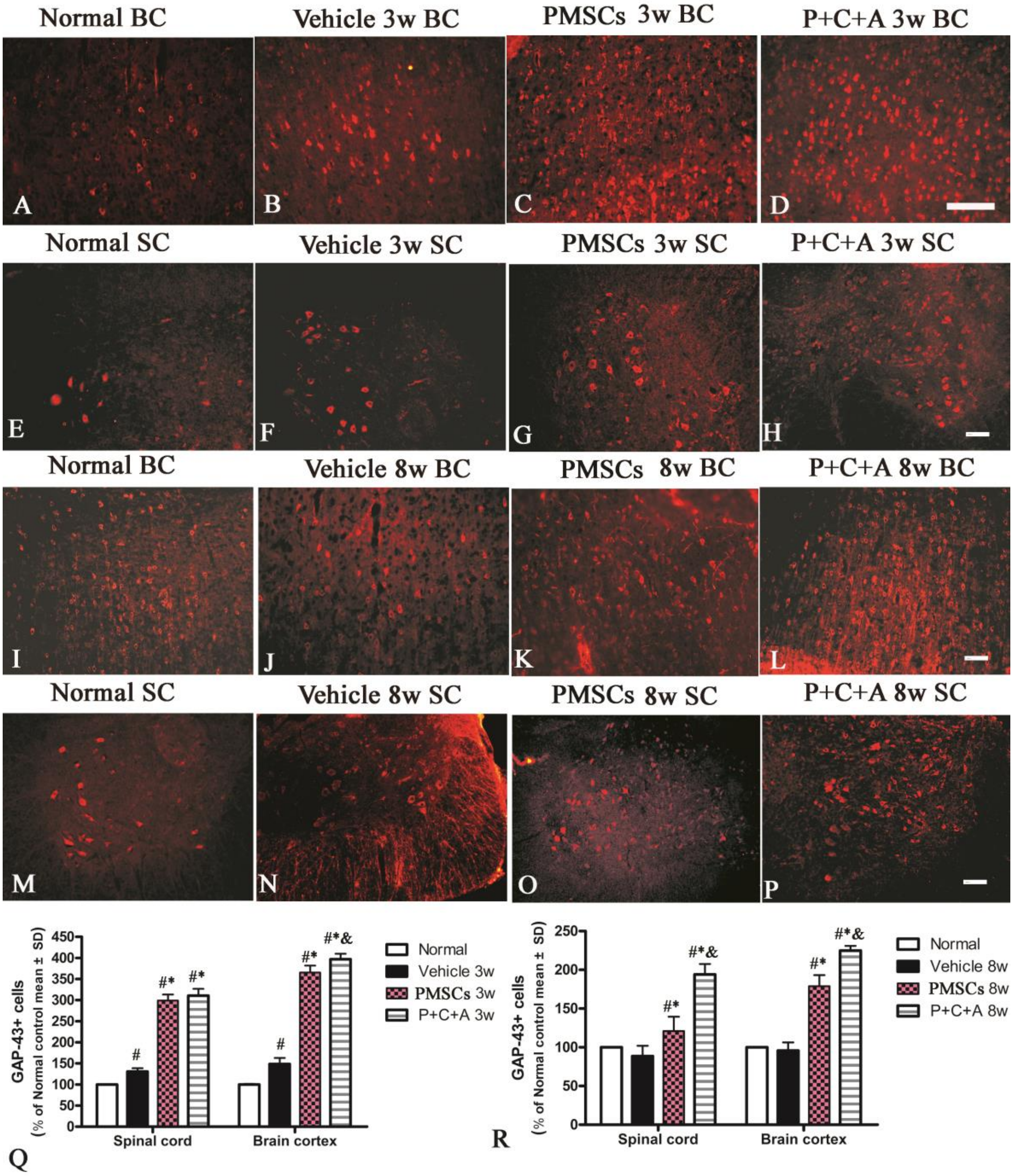
Normal BC
Vehicle 3w BC
PMSCs 3w BC
P+C+A 3w BC
A
B
C
D
Normal SC
Vehicle 3w SC
PMSCs 3w SC
P+C+A 3w SC
E
F
G
H
Normal BC
Vehicle 8w BC
PMSCs 8w BC
P+C+A 8w BC
I
J
K
L
Normal SC
Vehicle 8w SC
PMSCs 8w SC
P+C+A 8w SC
M
N
O
P
GAP-43+ cells
(% of Normal control mean ± SD)
Spinal cord
Brain cortex
Normal
Vehicle 3w
PMSCs 3w
P+C+A 3w
Q
R
Vehicle 8w
PMSCs 8w
P+C+A 8w

Figure S9

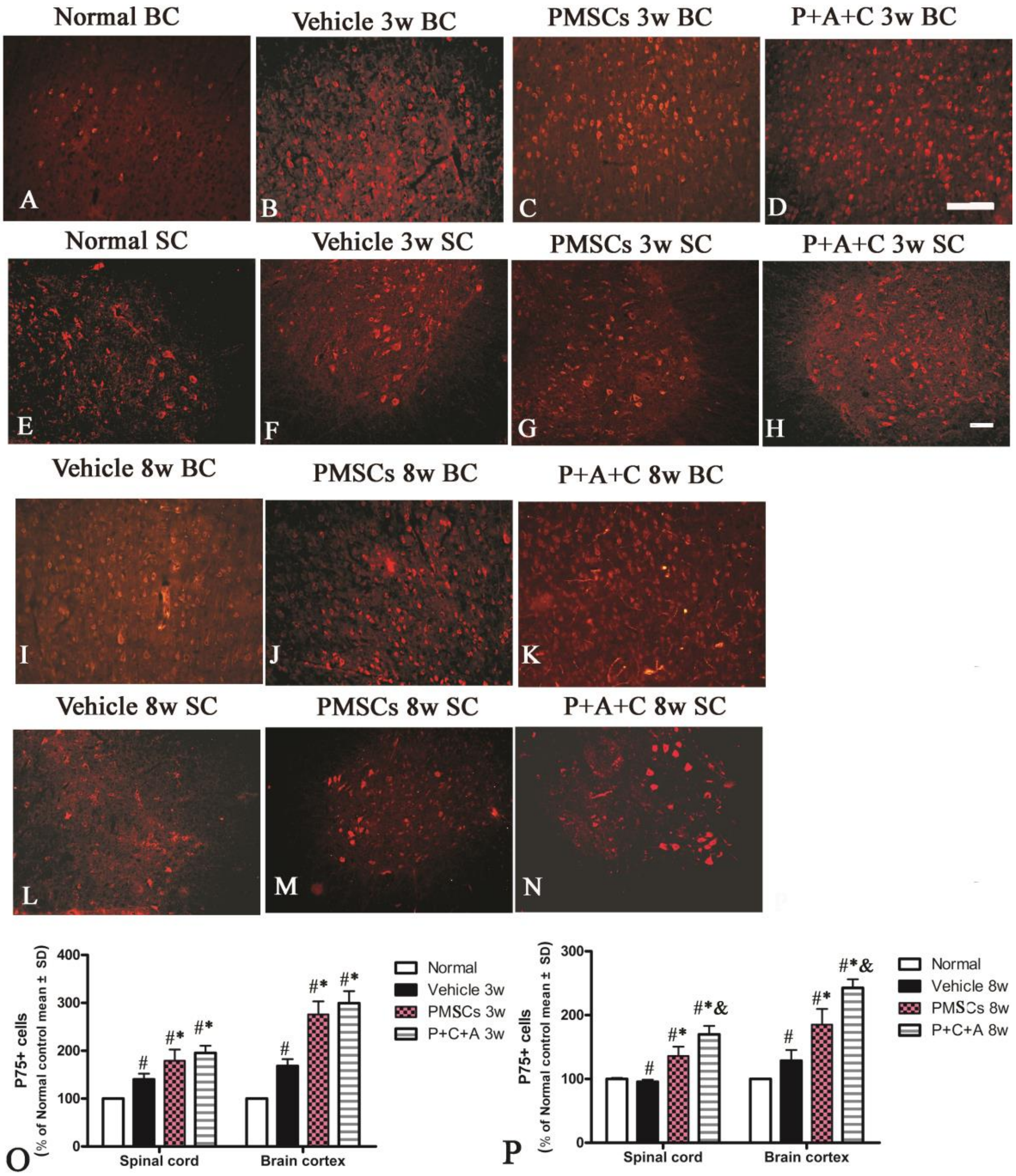

Normal BC
Vehicle 3w BC
PMSCs 3w BC
P+A+C 3w BC
A
B
C
D
Normal SC
Vehicle 3w SC
PMSCs 3w SC
P+A+C 3w SC
E
F
G
H
Vehicle 8w BC
PMSCs 8w BC
P+A+C 8w BC
I
J
K
Vehicle 8w SC
PMSCs 8w SC
P+A+C 8w SC
L
M
N
P75+ cells
(% of Normal control mean ± SD)
Spinal cord
Brain cortex
Normal
Vehicle 3w
PMSCs 3w
P+C+A 3w
O
Vehicle 8w
PMSCs 8w
P+C+A 8w
P

Figure S10

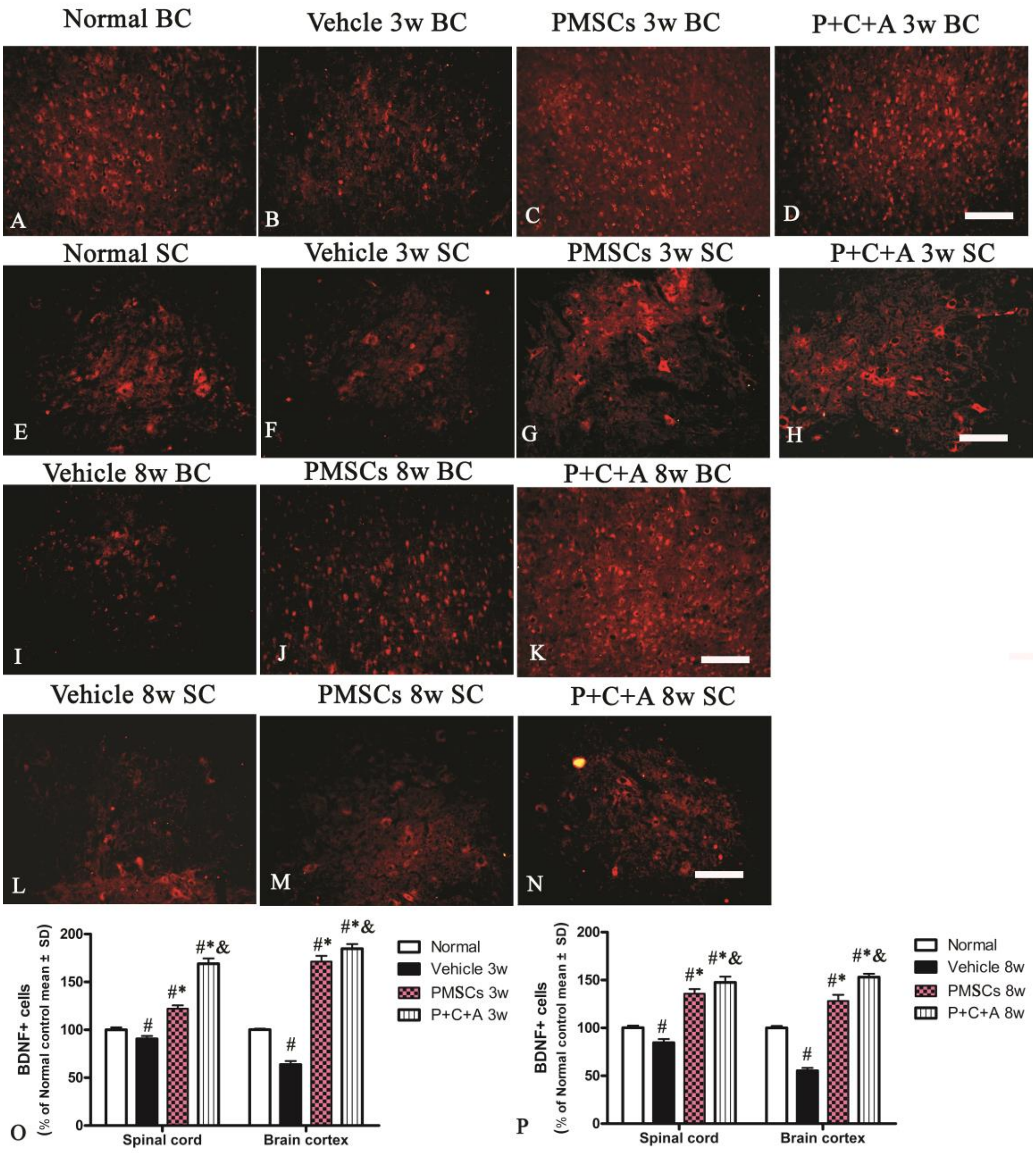

Normal BC
Vehcle 3w BC
PMSCs 3w BC
P+C+A 3w BC
A
B
C
D
Normal SC
Vehcle 3w SC
PMSCs 3w SC
P+C+A 3w SC
E
F
G
H
Vehicle 8w BC
PMSCs 8w BC
P+C+A 8w BC
I
J
K
Vehicle 8w SC
PMSCs 8w SC
P+C+A 8w SC
L
M
N
BDNF+ cells
(% of Normal control mean ± SD)
200
150
100
50
0
#
#*
#*&
Spinal cord
Brain cortex
Normal
Vehicle 3w
PMSCs 3w
P+C+A 3w
O
P
Vehicle 8w
PMSCs 8w
P+C+A 8w

Figure S11

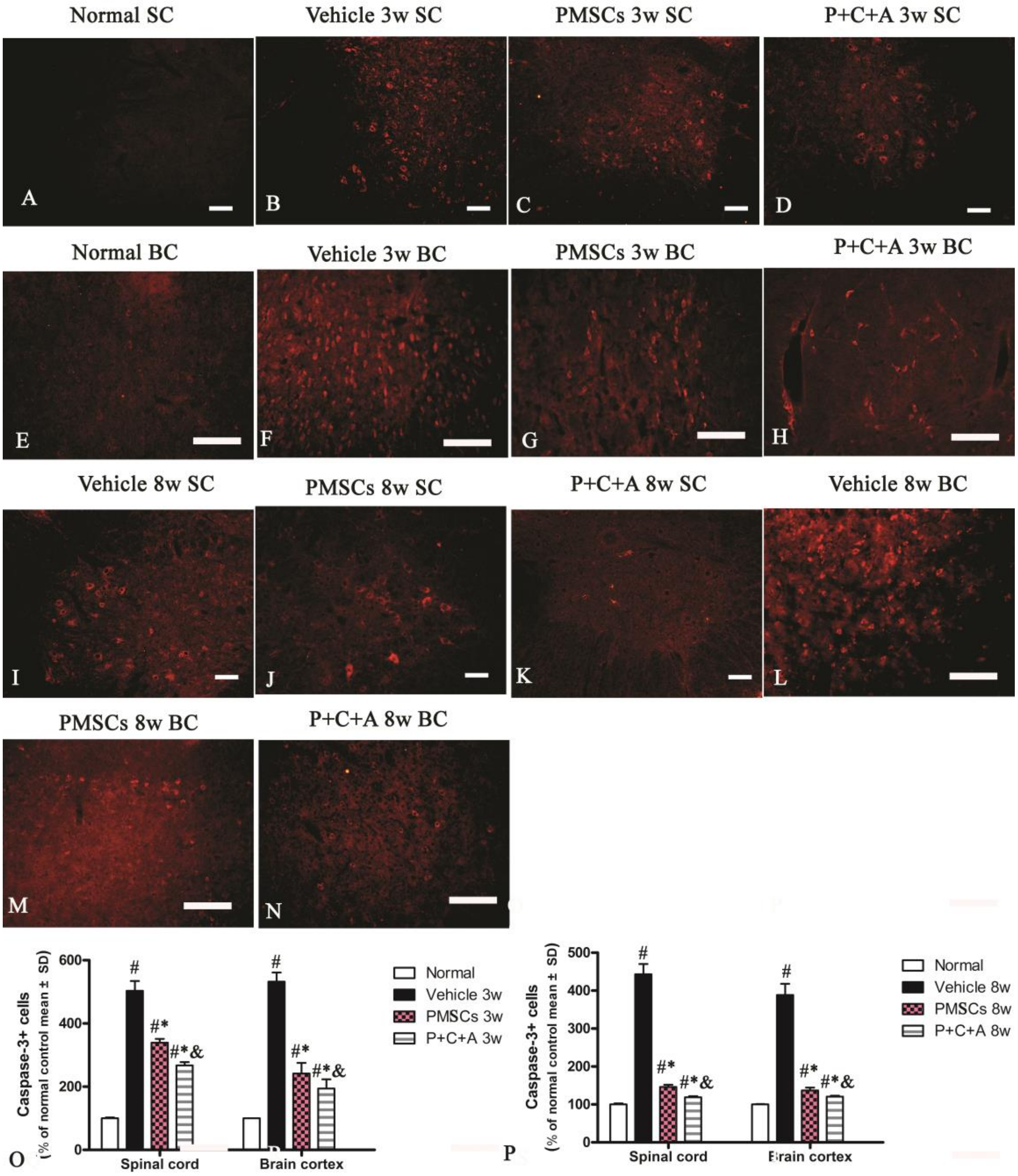
Normal SC
Vehicle 3w SC
PMSCs 3w SC
P+C+A 3w SC
A
B
C
D
Normal BC
Vehicle 3w BC
PMSCs 3w BC
P+C+A 3w BC
E
F
G
H
Vehicle 8w SC
PMSCs 8w SC
P+C+A 8w SC
Vehicle 8w BC
I
J
K
L
PMSCs 8w BC
P+C+A 8w BC
M
N
Caspase-3+ cells
(% of normal control mean ± SD)
600
400
200
0
Spinal cord
Brain cortex
Normal
Vehicle 3w
PMSCs 3w
P+C+A 3w
O
P
500
300
100
Normal
Vehicle 8w
PMSCs 8w
P+C+A 8w

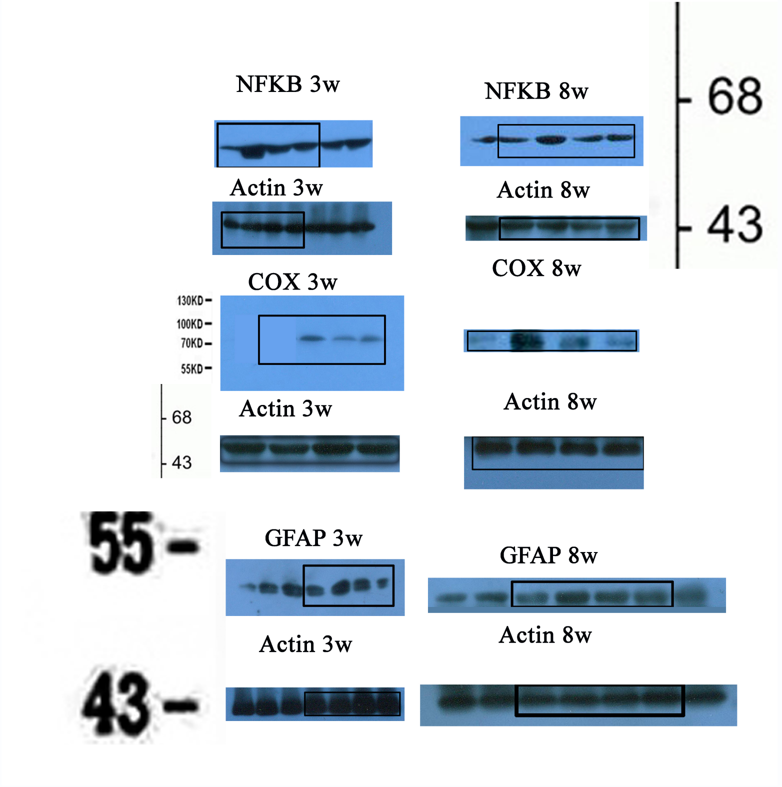

Supplementary Figure 12. Intravenous C16 and Ang-1 enhanced the anti-inflammatory and anti-astrogliosis effects of PMSCs in the brain cortex of rats with EAE.These are uncropped immunblot images corresponding to data shown in Figure 2 A-B, D-E, and G-H respectively.

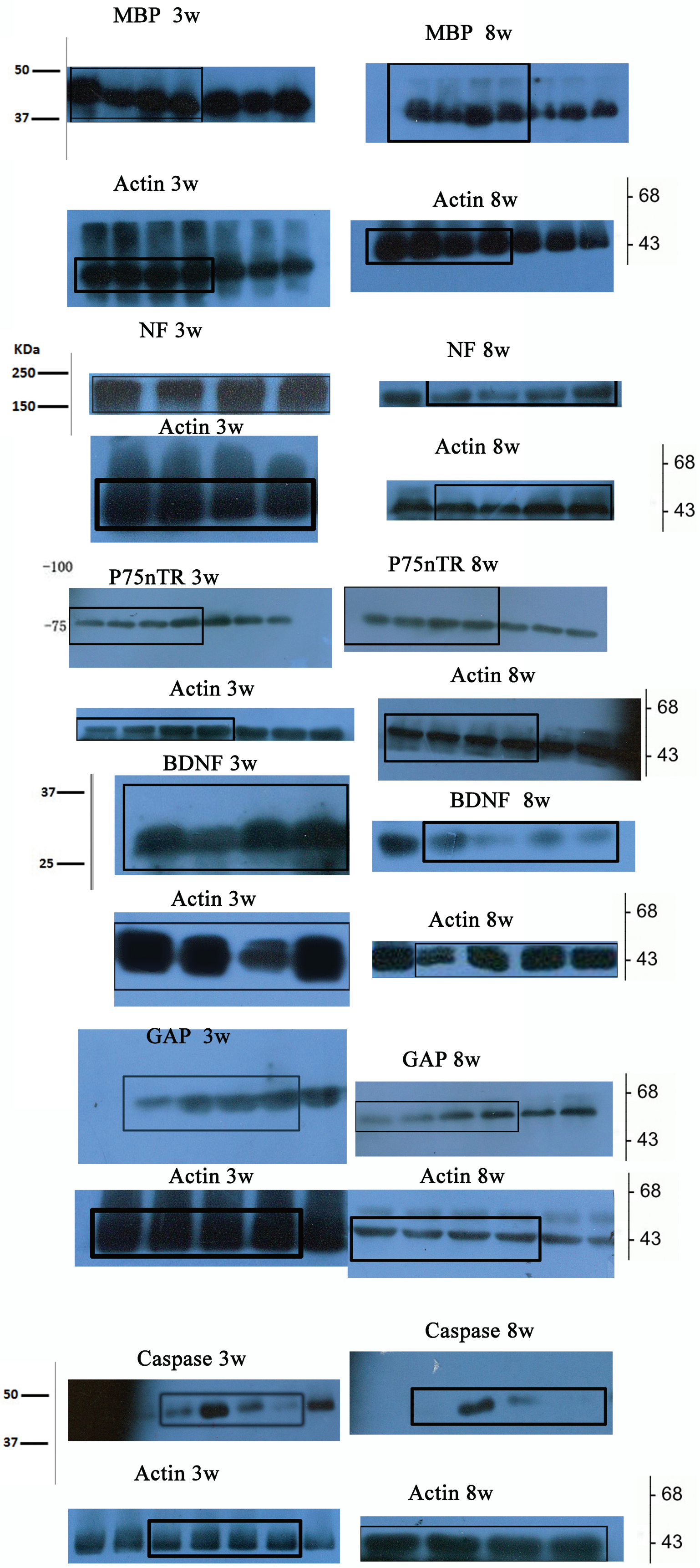

Supplementary Figure 13. There are uncropped immunblot images corresponding to data shown in Figure 5 to exhibited Protein expression of MBP (A-B), NF-200 (D-E), p75NTR (G-H), BDNF (J-K), GAP-43 (M-N) and caspase-3 (P-Q) in the brain cortex at 3 and 8 weeks pi by western blotting.
